# Supplementary material for: Genome-Wide Identification of CYP72A Gene Family and Expression Patterns Related to Jasmonic Acid Treatment and Steroidal Saponin Accumulation in Dioscorea zingiberensis
Source: Int J Mol Sci. 2021 Oct 11;22(20):10953. doi: 10.3390/ijms222010953 (PMC8536171; doi:10.3390/ijms222010953)
Supplement: Supplementary file 1 [file ijms-22-10953-s001.zip › Table S1 The amino acid sequences of DzCYP72A gene family.pdf]

The amino acid sequences of *DzCYP72A* gene family

>DzCYP72A1

MESVMGVVWAAAAVAVVAWAWRTLDWVWWKPRRLDRELRRQGLRGNQYRLLHGDLKENA
RLSEKAKSRPLPLHCHDIAPRVLPVLHNAIKDHGKISITWFGPYPRVTLMEPELVKEVLSNKFGH
FAKIRATPLANFLVQGLVTYEGEKWAKHRRIINPAFHLEKLKLMLPAFSTSCGELIRRWEKMIPD
EGSQELKCLSRAPRPHKRCHLQDCIRSSYEEGRRIFELLAEQIQLLIPAFQTIYIPGYRFLPTPMN
KRRSQVYNEMKRILKGMIEKREKAIRMGDSSKNDLLGLLLDSNMKEGEEHGKSQNKGMSTE
DVIEECKLFYFAGQETTAVLLTWTMILLSMYPNWQAKAREEVLQVFGKNTPDMEGLSHLKIVT
MILYEVLRLYPPFVLLRRKTYKAMELGGIIYPPGVMLSLPLLFIHHDPAFWGEDAKEFNPERFSE
GISKASKVPGAFFPFGGGPRICIGQSFAMIEAKIGICMILQCFSFELSPSYIHAPHTVITLQPQHGA
QLMLQKL

>DzCYP72A2

MESVMGVVWAAAAVAVAVVAWAWRTLDWVWWTPRRLDRELRQQGLRGNQYRVLHGDLKE
NARLSEEAKSRPLPLHCHDIAPRVVPVLHNAIKDHGKISITWFGPYPRVTLMEPELVKEVLSNKF
GHFAKVRPNPLTKLLVQGLVVYEGEKWAKHRRIINPAFHLEKLKLMLPAFSTSCGELIRRWEKM
IPDEGSHELNVFPELQDLTKDVISRTAFGSSYEEGRRIFELLAEQIQLLIPAFQTIYIPGYRFLPTPM
NKRRSQVYNEMKRILKGMIEKREKAIRMGDSSKNDLLGLLLDSNMKEGEEHGKSQNKGMST
EDVIEECKLFYFAGQETTAVLLTWTMILLSMYPNWQAKAREEVLQVFGKSTPDMEGLNHLKIV
TMILYEVLRLYPPFVFLTRKTYKAMELGGITYPQGVILSLPLLFIHHDPAFWGEDAKEFNPERFS
EGISKASKVPGAFFPFGGGPRICIGQSFAMIEAKIGICMILQRFSFELSPSYIHAPHTVITLQPQHG
AQLMLQKL

>DzCYP72A3

MELVMGVVWAAVAVVAWAWRTLDWVWWTPRRLDRELRRQGLRGNQYRLLHGDLKENAQL
SDEAKSRPLPLHCHDIAPRVLPLFHNAIKDHGKISITWLGPYPRVILAEPELVKEVLSNKFGHFV
KPSTTPLAKFLVQGLASYDGEKWVKHRRIINPAFHFEKLKQMLPAFSTSCGELIRRWNKMIPDE
GSQELNVFPELQGLTKDIISRTAFGSSYEEGRRIFELLTEQIKLTIPAFKTVYIPGYRFLPTPMNKR
RSQVYNEMKRILKGMIEKREKAIRMGESSKNDLLGLLLDSNMQEGEEHGKSQDKGMSTEDVI
EECKLFYIAGQETTSALLTWTMILLSMYPNWQANAREEVLQVFGKSTPDIEGLSHLKIVTMILY
EVLRLYPPGVFLDRKTYKAMELGGITYPSGVILSLPLLFIHNDPTFWGEDAKEFNPERFSEGISK
ASKVPGAFFPFGGGPRICIGQNFALMEAKIGICMIIQHFSFVLSPSYIHAPHSVITLQPQHGAQLM
LQKL

>DzCYP72A4

MELVMGVVWAAAVVMLVAWAWRTLDWVWRTPMRLDRELRRQGLRGNQYRVFHGDLKENA
RLSKEAESRPLPLHCHDIAPRVLPLFHNAIKDHGKISITWLGPCPRVTLTEPELVKEVLNKFGHF
VKPSTTPFAKFLVQGLVYYEGEHWAKHRRILNPAFHLEKLKLMLPAFSTSCSELIRRWEKMIPD
EGSQELNVFPELQGLTKDVISRTAFSSSYEEGRRIFELLKEQIQLYIQVYKTVYIPGYRFLPTPMN
KRRSQVYNEMKRILKGMIGEERKGHKNGESCKDDLLGLLLDSNMKEGEEHGKSQNKGMSTE
EVIEECKLFYFAGQETTPSLLLTWTMILLSMYPNWQTKAREEVLKVFGKNTPDMEGLSHLKIV
TMILYEVLRLYPPVVFITRKTYKAMELGGITYPPGVIFSLPLLFIHHDPTFWGEDAKEFNPERFSE
GISKASKVPAAFFPFGGGPRICIGQNFALIEAKIGICMILQHFSFVLSLLSYIHAPHTVITLQPEHG
AQLMLQKL

>DzCYP72A5

MESVMGVVWAAAAVAVVAWAWRTLDWVWWTPRRLDRELRQQGLRGNQYRVLHGDLKENT
RLSEEAKSRPLPLHCHDIAPRVVPVLHNAIKDHGKISITWFGPYPRVTLMEPELVKEVLSNKFGH

FAKVRPNPLTKLLVQGLVVYEGEKWAKHRRIINPAFHLEKLKLMLPAFSTSCGELIRRWEKMIP
DEGSHELNVFPELQDLTKMSSPGLHSVAAMKKEGRRIFELLAEQIQLLIPAFQTLYIPGYRFLPTP
MNKRRSQVYNEMKRILKGMIEKREKAIRMGEGSKNDLLGLLLDSNIKESEEHGKSQNKVMST
EDVIEECKLFYFAGQETTSLLLTWTMILLSMYPNWQAKAREEVLQVFGKNTPDMEGLSHLKIV
TMILYEVLRLYPPFVLLRRKTYKAMELGGITYPPGVILSLPLLFIHHDPAFWGEDAKEFNPERFS
EGISKASKVPGAFFPFGGGPRICIGQSFAMIEAKIGICMILQCFSFELSPSYIHAPHTVITLQPQHG
AQLMLQKL

>DzCYP72A6

MELVMGVVWAAVAVVAWAWRTLDWVWWTPRRLDRELRRQGLRGNQYRVLHGDLKENAQL
SEEAKSRPLPLHYHDIAPRVLPLFHNAIKDHEPELVKEVLSNKFGHFVKPSTTPLAKFLVQGLAS
YDGEKWVKHRRIINPAFHFEKLKQMLPAFSTSCGELIRRWNKMIPDEGSQELNVFPELQGLTKD
IISRTAFGSSYEEGRRIFELLTEQIKLTIPAFKTVYIPGYRFLPTPMNKRRSQVYNEMKRILKGMIE
KREKAIRMGESSKNDLLGLLLDSNMQEGEEHVKSQDKGMSTEDVIEECKLFYIAGQETTSALL
TWTMILLSMYPNWQANAREEVLQVFGKSTPDIEGLSHLKIVTMILYEVLRLYPPGVFLDSKTSK
AMELGGITYPSGVILSLPLLFIHNDPTFWGEDAKEFNPERFSEGISKASKVTGAFFPFGGGPRICI
GQNFALMEAKIGICMIIQHFSFVLSPSYIHAPHSVITLQPQHGAQLMLQKL

>DzCYP72A7

MRLDRELRRQGLRGNQYRVFHGDLKENARLSKEAESRPLPLHCHDIAPRVLPLFHNAIKDHGK
ISITWLGPCPRVTLTEPELVKEVLNKFGHFVKPSTTPFAKFLVQGLVSYEGEKWAKHRRILNPAF
HLEKLNLMLPAFSTSCSELIRRWEKMIPDEGSQELNVFPELQGLTKDVISRTAFSSSYEEGRRIFE
LLKEQIQLYIQVYKTVYIPGYRFLPTPMNKRRSQVYNEMKRILKGMIEKREKAIRMGESCKDD
LLGLLLDSNMKEGEEHGKSQNKGMSTEEVIEECKLFYFAGQETTSLLLTWTMILLSMYPNWQT
KAREEVLKVFGKNTPDMEGLSHLKIVTMILYEVLRLYPPVVFITRKTYKAMELGGITYPPGVIF
SLPLLFIHHDPTFWGEDAKEFNPERFSEGISKASKVPAAFFPFGGGPRICIGQNFALIEAKIGICMI
LQHFSFVLSLSYIHAPHTVITLQPEHGAQLMLQKL

>DzCYP72A8

MESVMGVVWAAAAVAVVAWAWRTLDWVWWKPRRLDRELRRQGLRGNQYRLLHGDLKENA
RLSEEAKSRPLPLHCHDIAPRVLPVLHNAIKDHGKISITWFGPYPRVTLMEPELVKEVLSNKFGH
FAKIRATPLANFLVQGLVTYEGEKWAKHRRIINPAFHLEKLKLMLPAFSTSCGELIRRWEKMIPD
EGSQELNVFPELQDLTKDVISRTAFGSSYEEGRRIFELLAEQIQLLIPAFQTIYIPGYRFLPTPMNK
RRSQVYNEMKRILKGMIEKREKAIRMGEGSKNDLLGLLLDSNIKESEEHGKSQNKVMSTEDVI
EECKLFYFAGQETTSLLLTWTMILLSMYPNWQGKAREEVLQVFGKSTPDMEGLNHLKIVTMIL
YEVLRLYPPFVFLTRKTYKAMELGGITYPQGVILSLPLLFIHHDPAFWGEDAKEFNPERFSEGIS
KASKVPGAFFPFGGGPRICIGQSFAMIEAKIGICMMLQRFSFELSPSYIHAPHTVITLQPQHGAQL
MLQKL

>DzCYP72A9

MESVMGVVWAAAAVAVAVVAWAWRTLDWVWWTPRRLDRELRQQGLRGNQYRVLHGDLKE
NARLSEEAKSRPLPLHCHDIAPRVVPVLHNAIKDHGKISITWFGPYPRVTLMEPELVKEVLSNKF
GHFAKVRPNPLTKLLVQGLVVYEGEKWAKHRRIINPAFHLEKLKLMLPAFSTSCGELIRRWEKM
IPDEGSHELNVFPELQDLTKDVISRTAFGSSYEEGRRIFELLAEQIQLLIPAFQTIYIPGYRFLPTPM
NKRRSQVYNEMKRILKGMIEKREKAIRMGDSSKNDLLGLLLDSNMKEGEEHGKSQNKGMST
EDVIEECKLFYFAGQETTAVLLTWTMILLSMYPNWQGKAREEVLQVFGKSTPDMEGLNHLKIV
TMILYEVLRLYPPFVFLTRKTYKAMELGGITYPQGVILSLPLLFIHHDPAFWGEDAKEFNPERFS
EGISKASKVPGAFFPFGGGPRICIGQSFAMIEAKIGICMILQRFSFEL

>DzCYP72A10
MELVMRVVWAVAAVAVVAWAWRTLDWVWWTPRRLDRELRRQGLRGNQYRLLHGDLKENAQ
LSEEAKSRPLPLHYHDIAPRVLPLFHNAIKDHGKISITWLGPYPRVILTEPELVKEVLSNKFGHFV
KPSTTPLAKFLVQGLASYDGEKWVKHRRIINPAFHFEKLKQMLPAFSTSCGELIRRWNKMIPDE
GSQELNVFPELQGLTKDIISRTAFGSSYEEGRRIFELLTEQIKLTIPAFKTVYIPGYQFLPTPMNKR
RSQVYNEMKRILKGMIEKREKAIRMGESSKNDLLGLLLDSNMQEGEEHGKSQDKGMSTEDVI
EECKLFYIAGQETTSALLTWTMILLSMYPNWQANAREEVLQVFGKSTPDIEGLSHLKIVTMILY
EVLRLYPPGVFLDRKTYKAMELGGITYPSGVILSLPLLFIHNDPTFWGEDAKEFNPERFSEGISK
ASKVPGSFLPYGVGPRICIGQNFALMEAKIGICMIIQHFSFVLSPSYIHAPHSVITLQPQHGAQLM
LQKL
>DzCYP72A11
MELVMGVVWAAAVVMLVAWAWRTLDWVWRTPMRLDRELRRQGLRGNQYRVFHGDLKENA
RLSKEAESRPLPLHCHDIAPRVLPLFHNAIKDHGKISITWLGPYPRVTLTEPELVIEVLNKFGHFV
KPSTTPFAKFLVQGLVSYEGEKWAKHRRILNPAFHLEKLKLMLPAFSTSCSELIRRWEEKIPDEG
SQELNVFPELQGLTKDVISRTAFSSSYEEGRRIFELLKEQIQLYIQVYKTVYIPGYRFLPTPMNKR
RSQVYNEMKRILKGMIEKREKAIRMGESCKDDLLGLLLDSNMKEGEEHGKTQNKGMSTEEVI
EECKLFYFAGQETTSLLLTWTMILLSMYPNWQAKAREEVLQVFGKNTPDMEGLSHLKIVTMIL
YEVLRLYPPVVFLTRKTYKAMELGGITYPPGVIFSLPLLFIHHDPTFWGEDAKEFNPERFSEGIS
KASKVSAAFFPFGGGPRICIGQNFALIQAKIGICMILQHFSFVLSLSYIHAPHTVITLQPQHGAQL
MLQKL
>DzCYP72A12
MESLMGVIWAVAAVVVVAAAWRTLDWIWWTPRRLDRELRRQGLRGNQYRVLHGDLKENVR
LSKEAKSRPLPLHCHDIAPRVLPLFHNAIKDHGKISITWLGPYPRVTLTEPELVKEVLSNKFGHF
VKPTTTPLAKFLVQGLVSYEGEKWAKHRRIINPAFHLEKLKLMLPAFSTSSGELIRRWEKMIPNE
GSQELNVFPELQDLTKDVISRTAFGSSYEDGRRIFELLTEQIQLLIPAFQTVYIPGFRFLPTPMNKR
SSQVYNEMKRILIGMIEKREKAIRMGESNKNDLLGLLLDSNMKEGEEHRKSQNKGMSTEDVIE
ECKLFYFAGQETTSVLLTWTMILLSMYPNWQAKAREEVLQVFGKSAPDMEGLSHLKIVTMILY
EVLRLYPPAVFLTRKTYKAMELGGITYPPGVILSLPLLFIHHDPVFWGEDAKEFNPERFSEGISK
ASKVPGAFFPFGGGPRICIGQNFALIEAKIGICMILQHFSFVLSPSYIHAPHNVITLQPQHGAQLM
LQKL
>DzCYP72A13
MELVMGVIWTVTAAVVVVWAWRTLDWVWWTPRRLDRDLRRQGLRGNQYRLLHGDLKENA
RLSKEATSRPLPLHCHDIAPRVAPLIHNAIKDHGKISITWLGPYPRVSLMDPDLVKEVLSNKFGH
FVKPRITPIAKFLVQGLVAYEGEKWAKHRRIINPAFHLEKLKLMLPAFSTSSGELIRRWEKMIPDE
GSQELNVSPEIQNLTGDVISRTAFGSSYEEGRRIFELLTEQIHLTIPALQTVYIPGYRFLPTPMNNR
RSQVYNEMKRILKGMIEKREKAIRMGESSKNDLLGLLLDSNMKEGEEQEKSQNKVMSTEDVI
EECKLFYFAGQETTSVLLTWTMILLSMYPNWQAKAREEVLQVFGKSTPDMDGLSRLKIVTMIL
YEVLRLYPPVTFLTRKTYKAMELGGINYPPGVLLQLPLLFIHHDPEFWGEDAKEFNPERFSEGIL
KASKVPGAFFPFGGGPRICIGQSFALIEAKIGISMILQHFSFVLSPSYIHAPHTALTLQPQHGAQL
MLQKL
>DzCYP72A14
MEYSVAAGVKEMIWGVATALLVVWVWRTLEWLWWKPRRLERELKRQGLRGGKYRLFHGDL
KYNARLMKDALSRPLPPYSHDVAPRVIPLVHQAIKIHGKMSITWLGPYPRVSLMDPELIREVLS
NKFGHFVKPNFSPLVKLLAQGLASHEGEKWAKHRRIINPAFHFEKLKCMLPAFSTCCDELVKR

WQNKMNVEGSIELNMWPELQNLTGDVISRTAFGSCYEEGRRIFQLQTEQAELIIPTAQTVYVPG
FRFLPTPKNNRRKAIDREIKTILRSMIEKREKAIRMGGETSCKDDLLGLLLESNMKETEQQGRS
KNKGLTTEDVIEECKLFYLAGQETTSSLLTWTMVLLSMYPNWQAKAREEVLQVFGKNKPDM
DGVGRLKIVTMILYEVLRLYPPAISLTRRTYKTMELGGITYPPGVLLLLPIILVHHDLDFWGEDA
KEFKPDRFAEGISKASKVPGAFFPFGGGPRICIGQSFALIEAKLGLSMILQNFSFELSPSYIHAPYT
LVTLQPQHGAPIKLHKL

>DzCYP72A15

MEPSFISMAATLSSLLLLYCALTVVHVVWWRPRMIEKQLKKQGIKGRPYKVLRGDLSDIVKIM
KEALSKPMELHHHISPRALPFVHSTVEQFGKLSIIWYGKNPRIIIQDVELIKEVLANKNGNFLKP
LLNPLQRLLAEGVSLVEGDKWVQHRNILNPAFHLAKLKGMVPAFCTSCSEMISKWEMLFGPE
GSCELDVWIELKALTADVISRTAFGSNYREGQKVFEFQQEQIQLMMEASWIPYIPGFRFLPTKK
NRRRYYLDNEIKAIIRSLIHKKEKSMEVGESGGEDLLSLLLQSNHNVHENAANGLKLEGLTIDE
VIEECKLFYFAGHDTTSSLLTWTLILLSMYPAWQTRAREEVHRICGKNMPDYESIGQFKIVTMIL
HEVLRLYPPVTGQYRHVYHETKLGELSLPAGVDLFVPSLLVYHDPEIWGEDCKEFNPQRFSEG
VLKATKNQFVYFPFGFGQRTCIGQNFAMIEAKIALAMMLQHFSFDLSPSYAHCPYSLITLQPQH
GAQLIVHRL

>DzCYP72A16

MVVVVVFVSLVVIVGLVARVSWWIWWKPKMMEKEMRRQGINGTKYRTFMGDLKDEGEAYK
EAWSKPMDLNHNIVPRVLPHAHLMLQTYGKTSFKWMGTTPAVTIWEPELLREVLMNKCGHFV
QPQVNPLIKLLANGINSLEGEQWAQRRKMIKPAFHIDKLKEMVPTFITSCSELIERWKKLLTDET
SCELDVWPEFQNLTIDAISRILFGNSFEEGKRIFELQKEQIVLAIDAASSIYVPGFRFLPTTKNKRR
MFIDNEIKRILQDIINNKLESMKMGGNANSDILSLMLQYGNINIAEKEKNTNNSKITIDNVIEEC
KGFYFAGQDTTSTLLTWTLILLSMHPTWQQKAREEVLRTCGKNTPNFESINHLKIVNMILHEVL
RLYPPVITQIRHTEKKTKLGDITLPAGVHVLIPTLQVHHDQEFWGEDAEEFNPERFSEGVSKASK
GQNAFLPFGWGPRMCLGQAFAMIETKLGLAMVLQHFSFELSPSYAHAPSIVATLQPQLGAHLIL
HQL

>DzCYP72A17

MLEMQLRRQGLPGNKYRLMMGDMKDEKKSFKEAWSRPMELTHRIAARVIPYDHQMAQTHG
KISFKWNGTTPRVNIWNPEMSREILLNKSGHIIKPQLNPLIRLLTMGVSTLEGEEWAQRRKLINP
AFHMEKLKEMVPVFRISCIDLVKRWENLVSAEGSCELDVWPEFQSLTGDVISRTAFGSSFEEGK
QIFELQKEQAVLVIEASRSIYLPGFRFLPTAKNKRRMFIDSEIKRMLRDIIHKKLDSMKIGENADD
DLLSLLLQSDTMNVVAEDKNKKNNGITIDDVIEECKLFYFAGQEGTSILLTWTLILLSMYPSWQ
KKAREEVLNNCGKNTPEFENISHLKIVNMILHEVLRLYPPGVTLIRYINKKVKVGNITLPAGAEV
LIPILQVHHDPEIWGEDAEEFKPERFSEGVSNASKGQQAFFPFGWGPRICSGQTFAMIEAKLAL
AMVLQNFSFELSPSYTHAPYNVITLQPQYGAHLILHQL

>DzCYP72A18

MNVATEDKNKKNNGITIDDVIEECKLFYFAGQETTSILLTWTLILLSMYPTWQKKAREEVLNTC
GKNTPEFENISHLKIVNMILHEVLRLYPPVITLFRHINKNVKLGDITLPAGAEVLIPILQVHHDPEI
WGEDAEEFKPERFSEGVSNASKGQNAFFPFGWGPRILIGVLGRVSYLVWWKPKDAGDAAEKT
GVARNKYRLMMGDMKDEKKSFKEAWSRPMELTHRIAARVIPYDHQMAQTHGKISFKWNGTT
PRVNIWNPEMSREILLNKSGHIIKPQLNPLIRLLTMGVSTLEGEEWAQRRKLINPAFHMEKLKE
MVPAFRISCIDLVKRWENLVSAEGSCELDVWPEFQSLTGDVISRTAFGSSFEEGKQIFEFQKEQA
VLVIEAARSIYLPGFRFLPTAKNKRRMFIDSEIKRMLRDIIHKKIDSMKIGENTDDDLLSLFICNPI
L

>DzCYP72A19
MYPTWQKKAREEVLNTCGKNTPEFENVSHLKIVNMILHEVLRLYPPVITLFRHINKNVKLGDIT
LPAGAEVVIPILQVHHDPEIWGEDAEEFKPERFSEGVSNASKGQNAFFPFGWGPRICIGQTFAMI
EAKLALAMVLQHFSFDLSPSYTHAPYTVITLQPQYGAHLILHQL
>DzCYP72A20
MGVLWRVLYLVWWKPKMLEMQLKRQGLPGNKYRLMMGDMKDEKKSFKEAWSRPMELTHR
IAARVIPYDHQIMAQTHGKISFKWKGTTPRVNIWNPEMLKEILLNKSGHIIKPQVNPLIKLLTMG
VSTLEGEEWAQRRKLINPAFHMEKLKEMVPAFRISCIDLVKRWENLVSAEGSCELDVWPEFQSL
TGDVISRTAFGSSFEEGKQIFELQKEQAVLVIEAARSIYLPGFRFLPTAKNKRRMFIDSEIKRMLR
DIIHKKIDSMKIGENTDDDLLSLLLQSDTMNVATEDKNKKNNGITIDDVIEECKLFYFAGQETTS
ILLTWTLILLSMYPTWQKKAREEVLNTCGKNTPEFENISHLKIVNMILHEVLRLYPPVITLFRHIN
KNVKLGDITLPAGAEVLIPILQVHHDPEIWGRMLKSSKPERFSEGVSNASKGQNAFFPFGWGPR
ICIGQTFAMIEAKLALAMVLQHFSFDLSPSYTHAPYTVITLQPQYGAHLILHQL
>DzCYP72A21
MLEMQLRRQGLPGNKYRLMMGDMKDEKKSFKEAWSRPMELTHRIAARVIPYDHQMAQTHG
KISFKWNGTTPRVNIWNPEMSREILLNKSGHIIKPQLNPLIRLLTMGVSTLEGEEWAQRRKLINP
AFHMEKLKEMVPVFRISCIDLVKRWENLVVSAEGSCELDVWPEFQSLTGDVISRTAFGSSFEEG
KQIFELQKEQAVLVIEAARSIYLPGFRFLPTAKNKRRMFIDSEIKRMLRDIIHKKIDSMKIGENTD
DDLLSLLLQSDTMNVATEDKNKKNNGITIDDVIEECKLFYFAGQETTSILLTWTLILLSMYPTW
QKKAREEVLNTFGKNTPEFENISHLKIVNMILHEVLRLYPPVITLFRHINKNVKLGDITLPAGAE
VLIPILQVHHDPEIWGEDAEEFKPERFSEGVSNASKGQNAFFPFGWGPRICIGQTFAMIEAKLAL
AMVLQHFSFDLSPSYTHAPYTVITLQPQYGAHLILHQL
>DzCYP72A22
MLEMQLRRQGLPGNKYRLMMGDMKDEKKSFKEAWSRPMELTHRIAARVIPYDHQMAQTHG
KISFKWNGTTPRVNIWNPEMSREILLNKSGHIIKPQLNPLIRLLTMGVSTLEGEEWAQRRKLINP
AFHMEKLKEMVPVFRISCIDLVKRWENLVSAEGSCELDVWPEFQSLTGDVISRTAFGSSFEEGK
QIFELQKEQAVLVIEASRSIYLPGFRFLPTAKNKRRMFIDSEIKRMLRDIIHKKLDSMKIGENADD
DLLSLLLQSNTMNVAAEDKNKKNNGITIDDVIEECKLFYFAGQEGTSILLTWTLILLSMYPSWQ
KKAREEVLNNCRKNTPEFENISHLKIVNMILHEVLRLYPPGVTLIRYINKKVKVGNITLPAGAEV
LIPILQVHHDPEIWGEDAEEFKPERFSEGVSNASKGQQAFFPFGWGPRICSGQTFAMIEAKLAL
AMVLQNFSFELSPSYTHAPYNVMTLQPQYGAHLILHQL
>DzCYP72A23
MESVMGVIWAVAAVVVVAAAWRTLDWIWWTPRRLDRELRRQGLRGNQYRVLHGDLKENVR
LSKEAKSRPLPLHCHDIAPRVLPLFHNAIKDHGKISITWLGPYPRVTLTEPELVKEVLSNKFGHF
VKPTTNPLAKFLIQGLASYEGEKWAKHRRIINPAFHLEKLKLMLPAFSTSCGELIRRWEKMIPNE
GSQELNVFPELQDLTKDVISRTAFGSSYEDGRRIFELLTEQIQLLIPAFQTVYIPGYRFLPTPMNK
RSSQVYNEMKRILIGMIEKREKAIRMGESSKNDLLGLLLDSNIKESEVHGKSQNKGMSTEDVV
EECKLFYFAGQETTSLLLTWTMILLSMYPNWQAKAREEVLQVFGKSAPDMEGLSHLKIVTMIL
YEVLRLYPPAVFLTRKTYKAMELGGITYPPGVILSLPLLFIHHDPVFWGEDAKEFNPERFSEGIS
KASKVPGAFFPFGGGPRICIGQNFALIEAKIGICMILQHFSFVLSPSYIHAPHNVITLQPQHGAQL
MLQKV
>DzCYP72A24
MELVMGVIWTVTAAVVVVWAWRTLDWVWWTPRRLDRDLRRQGLRGNQYRLLHGDLKENA
RLSKEATSRPLPLHCHEIAPRVAPLIHNAIKDHGKISITWLGPYPRVSLMDPDLVKEVLSNKFGH

FVKPRITPIAKFLVQGLVAYEGEKWAKHRRIINPAFHLEKLKLMLPAFSTSSGELIRRWEKMIPDE
GSQELNVSPEIQNLTGDVISRTAFGSSYEEGRRIFELLTEQIHLTIPALQTVYIPGYRFLPTPMNNR
RSQVYNEMKRILKGMIEKREKAIRMGESSKNDLLGLLLDSNMKEGEEQEKSQNKVMSTEDVI
EECKLFYFAGQETTSVLLTWTMILLSMYPNWQAKAREEVLQVFGKSTPDMDGLSRLKIVTMIL
YEVLRLYPPVTFLTRKTYKAMELGGINYPPGVLLQLPLLFIHHDPEFWGEDAKEFNPERFSEGIL
KASKVPGAFFPFGGGPRICIGQSFALIEAKIGISMILQHFSFELSPSYIHAPHTPLTLQPQHGAQLM
LQKL

>DzCYP72A25

MEYSVAAGVKEMIWGVATALLVVWVWRTLEWLWWKPRRLERELKRQGLRGGKYRLFHGDL
KDNARLMKDALSRPLPPYSHDVAQRVIPLVHQAIKIHGKMSITWLGPYPRVSLMDPELIREVLS
NKFGHFVKPNFSPLVKLLAQGLASHEGEKWAKHRRIINPAFHFEKLKCMLPAFSTCCDELVKR
WQNKMNVEGSIELNMWPELQNLTGDVISRTAFGSCYEEGRRIFQLQTEQAELIIPTAQTVYVPG
FRFLPTPKNNRRKAIDREIKTILRSMIEKREKAIRMGGETSCKDDLLGLLLESNMKETEQQGRS
KNKGLTTEDVIEECKLFYLAGQETTSSLLTWTMVLLSMYPNWQAKAREEVLQVFGKNKPDM
DGVGRLKIVTMILYEVLRLYPPAIFLTRRTYKTMELGGITYPPGVLLLLPIILVHHDLDFWGEDA
KDFKPDRFAEGISKASKVPGAFFPFGGGPRICIGQSFALIEAKLGLSMILQNFSFELSPSYIHAPYT
LVTLQPQHGAPIKLHKL
