# Supplementary material for: Genome-Wide Identification of CYP72A Gene Family and Expression Patterns Related to Jasmonic Acid Treatment and Steroidal Saponin Accumulation in Dioscorea zingiberensis
Source: Int J Mol Sci. 2021 Oct 11;22(20):10953. doi: 10.3390/ijms222010953 (PMC8536171; doi:10.3390/ijms222010953)
Supplement: Supplementary file 1 [file ijms-22-10953-s001.zip › Table S2 The coding sequence (CDS) of DzCYP72A gene family .pdf]

The coding sequence (CDS) of *DzCYP72A* gene family

>DzCYP72A1

ATGGAGTCAGTGATGGGAGTGGTATGGGCGGCGGCGGTGGCGGTGGTGGCGTGGGCG
TGGAGGACGTTGGATTGGGTTTGGTGGAAGCCGAGGAGGCTGGATCGGGAACTCCGGCGG
CAGGGCCTGCGCGGCAACCAGTACCGACTCTTGCACGGCGATCTCAAGGAAAACGCCCGG
CTCTCCGAGAAGGCTAAATCCCGGCCTCTGCCTCTTCACTGCCATGACATCGCCCCCCGCGT
TCTCCCTGTCCTCCACAACGCCATCAAAGATCACGGTAAAATCTCAATAACTTGGTTTGGGC
CTTACCCAAGAGTGACCTTAATGGAGCCAGAGCTAGTCAAAGAAGTGTTGTCAAACAAATT
TGGACATTTTGCTAAAATAAGAGCAACTCCCCTTGCCAATTTTTTGGTCCAAGGACTTGTGA
CTTATGAAGGTGAAAAGTGGGCCAAACATAGAAGGATCATCAACCCTGCATTCCATCTTGA
GAAACTAAAGCTAATGCTTCCAGCATTCTCTACATCTTGTGGTGAACTGATTAGAAGATGGG
AGAAGATGATCCCTGATGAAGGCTCCCAAGAACTAAAATGTCTTTCCAGAGCTCCAAGACC
TCACAAAAGATGTCATCTCCAGGACTGCATTCGTAGCAGCTATGAAGAAGGAAGAAGAATA
TTTGAACTCCTAGCAGAGCAAATTCAGCTTCTTATCCCAGCTTTCCAGACTATATACATCCCT
GGTTATCGATTTCTGCCCACACCAATGAACAAAAGAAGAAGCCAAGTGTACAATGAGATGA
AAAGAATTCTTAAAGGCATGATAGAGAAGAGAGAGAAGGCCATAAGAATGGGGGACAGTA
GCAAGAATGACCTTCTGGGTTTGTTACTAGACTCCAATATGAAGGAGGGTGAAGAGCATGG
GAAGTCCCAAAATAAAGGGATGAGCACTGAGGATGTGATTGAAGAGTGCAAGCTGTTCTAC
TTTGCAGGGCAAGAGACTACAGCAGTTCTACTCACATGGACAATGATTTTATTGAGCATGTA
TCCAAACTGGCAGGCTAAGGCAAGAGAAGAGGTACTTCAAGTCTTTGGAAAGAACACACC
AGACATGGAGGGATTGAGCCACTTGAAGATTGTGACCATGATTCTATATGAAGTTCTGAGGT
TATATCCACCATTTGTTTTACTAAGAAGAAAAACCTACAAAGCAATGGAACTCGGTGGGATT
ATTTACCCTCCGGGAGTGATGCTCTCACTGCCTCTACTCTTCATTCACCATGACCCTGCTTTT
TGGGGAGAAGACGCCAAAGAGTTTAATCCAGAGAGGTTTTCCGAAGGGATATCAAAAGCA
TCCAAAGTTCCAGGTGCCTTCTTTCCTTTCGGTGGAGGTCCACGCATTTGCATTGGCCAAAG
CTTTGCAATGATTGAAGCTAAGATAGGAATTTGCATGATTCTTCAGTGTTTCTCCTTTGAGCT
TTCACCTTCCTATATCCATGCACCGCACACTGTTATTACTCTTCAACCACAGCATGGGGCTCA
ACTCATGCTGCAAAAGCTGTGA

>DzCYP72A2

ATGGAGTCAGTGATGGGAGTGGTATGGGCGGCGGCGGTGGCGGTGGCGGTGGTGGCG
TGGGCGTGGAGGACGTTGGATTGGGTTTGGTGGACGCCGAGGAGGCTGGATCGGGAGCTC
CGGCAGCAGGGCCTGCGCGGCAACCAGTACCGAGTCTTGCACGGCGATCTCAAGGAAAAC
GCCCGGCTATCCGAGGAGGCTAAATCCCGGCCTCTGCCTCTTCACTGCCATGACATCGCCCC
CCGCGTTGTCCCTGTCCTCCACAACGCCATCAAAGATCACGGTAAAATCTCAATAACTTGGT
TTGGGCCTTACCCAAGAGTGACCTTAATGGAGCCAGAGCTAGTGAAAGAAGTGTTGTCAA
ACAAGTTTGGACATTTTGCTAAAGTAAGACCAAATCCCCTTACCAAATTATTGGTCCAAGGA
CTTGTAGTTTATGAAGGTGAAAAGTGGGCCAAACACAGAAGGATCATCAACCCTGCATTCC
ATCTTGAGAAACTAAAGCTAATGCTTCCAGCATTCTCTACATCTTGTGGTGAACTGATTAGA
AGATGGGAGAAGATGATCCCTGATGAAGGCTCCCATGAACTAAATGTCTTTCCAGAGCTCC
AAGACCTCACAAAAGATGTCATCTCCAGGACTGCATTCGGTAGCAGCTATGAAGAAGGAAG
AAGAATATTTGAACTCCTAGCAGAGCAAATTCAGCTTCTTATCCCAGCTTTCCAGACTATATA
CATCCCTGGTTATCGATTTCTGCCCACACCAATGAACAAAAGAAGAAGCCAAGTGTACAAT
GAGATGAAAAGAATTCTTAAAGGCATGATAGAGAAGAGAGAGAAGGCCATAAGAATGGGG
GACAGTAGCAAGAATGACCTTCTGGGTTTGTTACTAGACTCCAATATGAAGGAGGGTGAAG

AGCATGGGAAGTCCCAAAACAAAGGGATGAGCACTGAGGATGTGATTGAAGAGTGCAAGC
TGTTCTACTTTGCAGGGCAAGAGACTACAGCAGTTCTACTCACATGGACAATGATTTTATTG
AGCATGTATCCAAACTGGCAGGCTAAGGCAAGAGAAGAGGTACTTCAAGTCTTTGGAAAG
AGCACACCAGATATGGAGGGATTGAACCACTTGAAGATTGTGACCATGATTCTATATGAAGT
TCTGAGGTTATATCCACCATTTGTTTTCCTAACAAGAAAAACCTACAAAGCAATGGAACTCG
GTGGGATTACTTACCCTCAAGGAGTGATACTCTCACTGCCTCTACTCTTCATTCACCATGACC
CTGCTTTTTGGGGAGAAGACGCCAAAGAGTTTAATCCAGAGAGGTTTTCCGAAGGGATATC
GAAAGCATCCAAAGTTCCGGGTGCCTTCTTTCCTTTCGGTGGAGGTCCGCGCATTTGCATTG
GCCAAAGCTTTGCAATGATTGAAGCTAAGATAGGAATTTGCATGATTCTTCAGCGCTTCTCC
TTTGAGCTTTCGCCTTCCTATATCCATGCACCGCACACTGTTATTACTCTTCAACCACAGCAT
GGGGCTCAACTCATGCTGCAAAAGCTGTGA

>DzCYP72A3

ATGGAGTTAGTGATGGGAGTGGTATGGGCGGCGGTGGCGGTGGTGGCGTGGGCGTGGAGG
ACGTTGGATTGGGTTTGGTGGACGCCGAGGAGGCTGGATCGGGAGCTCCGGCGGCAGGGC
CTGCGCGGCAACCAGTACCGACTCTTGCACGGCGATCTCAAGGAAAACGCCCAGCTCTCC
GATGAAGCTAAATCCCGGCCTCTGCCTCTTCACTGCCATGACATCGCCCCCCGCGTTCTCCC
TCTCTTCCACAACGCCATCAAAGATCACGGTAAAATCTCAATAACTTGGCTTGGTCCTTACC
CAAGAGTGATCTTAGCAGAGCCAGAGCTAGTGAAAGAAGTACTCTCAAACAAGTTTGGAC
ATTTTGTTAAACCAAGTACAACTCCCCTTGCCAAATTTTTGGTCCAAGGGCTTGCGTCTTAT
GACGGTGAAAAGTGGGTCAAACACAGAAGGATCATCAACCCTGCATTCCATTTTGAGAAA
CTAAAGCAAATGCTGCCAGCATTCTCTACGTCTTGTGGTGAACTTATTAGAAGATGGAACAA
GATGATCCCTGATGAAGGCTCCCAAGAACTAAATGTCTTTCCAGAGCTCCAAGGCCTCACA
AAAGATATCATCTCCAGAACCGCATTCGGTAGCAGCTATGAAGAAGGGAGAAGAATATTTG
AACTCCTAACTGAACAAATTAAGCTTACTATTCCAGCTTTCAAGACTGTATACATCCCTGGTT
ATCGATTTCTGCCCACACCAATGAACAAAAGAAGAAGCCAAGTGTACAATGAGATGAAAA
GAATTCTTAAAGGCATGATTGAGAAGAGAGAGAAGGCCATAAGAATGGGGGAAAGTAGCA
AGAATGACCTTCTGGGTTTGCTACTAGACTCCAATATGCAGGAGGGTGAAGAGCATGGGAA
GTCCCAAGACAAAGGGATGAGCACTGAGGATGTGATTGAAGAGTGCAAGCTGTTTTACATT
GCAGGGCAAGAGACTACATCAGCTCTACTCACTTGGACAATGATTTTATTGAGCATGTATCC
GAACTGGCAGGCTAATGCAAGAGAAGAGGTTCTTCAAGTCTTTGGAAAGAGCACACCAGA
CATAGAGGGATTGAGTCACTTGAAGATTGTGACCATGATTCTATATGAAGTTCTCAGGTTATA
TCCACCAGGGGTTTTTCTTGATAGAAAAACCTACAAAGCAATGGAACTTGGTGGGATTACT
TACCCTTCAGGAGTGATACTCTCACTACCTCTACTCTTCATTCACAATGACCCTACTTTCTGG
GGAGAGGATGCCAAAGAGTTCAATCCAGAGAGGTTTTCCGAAGGGATATCAAAAGCATCC
AAAGTTCCAGGGGCCTTCTTTCCTTTTGGTGGAGGTCCGCGCATTTGCATTGGCCAAAACTT
TGCACTGATGGAAGCTAAGATAGGAATTTGCATGATTATTCAGCACTTCTCATTTGTGCTTTC
ACCTTCCTATATCCATGCACCGCACTCTGTTATTACTCTTCAACCACAGCATGGAGCTCAACT
CATGCTGCAAAAGCTGTGA

>DzCYP72A4

ATGGAGTTAGTGATGGGAGTGGTATGGGCGGCGGCGGTGGTGATGCTGGTGGCGTGGGCGT
GGAGGACGTTGGATTGGGTTTGGCGGACGCCGATGAGGCTGGACCGGGAGCTCCGGCGCC
AGGGCCTGCGCGGCAACCAGTACCGAGTCTTCCATGGCGATCTCAAGGAAAACGCCCGGC
TCTCGAAGGAGGCTGAATCCCGGCCTCTGCCTCTTCACTGCCATGACATCGCCCCCCGCGTT
CTCCCTCTCTTCCACAATGCCATCAAAGATCACGGTAAAATCTCAATAACTTGGCTTGGCCC

TTGTCCAAGAGTGACCTTAACGGAACCAGAACTAGTAAAAGAAGTGTTAAACAAGTTTGG
ACATTTTGTTAAACCAAGTACAACTCCTTTTGCCAAATTTTTGGTCCAAGGGCTTGTGTATTA
TGAAGGAGAACACTGGGCCAAACATAGAAGGATACTCAACCCTGCATTCCATCTTGAGAAA
CTAAAGCTAATGTTGCCAGCATTCTCTACATCTTGTAGTGAATTGATTAGAAGATGGGAGAA
GATGATTCCTGATGAAGGCTCCCAAGAACTAAATGTCTTTCCAGAGCTCCAAGGCCTCACA
AAAGATGTCATCTCTAGGACTGCATTCAGTAGCAGCTATGAAGAAGGGAGAAGAATATTTG
AACTCCTAAAAGAGCAAATTCAGCTTTATATTCAAGTTTACAAGACTGTATACATCCCTGGTT
ATCGATTTCTGCCCACACCAATGAACAAAAGAAGAAGCCAAGTATACAATGAGATGAAAAG
AATTCTTAAAGGCATGATTGGAGAAGAGAGAAAAGGCCATAAGAATGGGGAAAGTTGTAA
GGATGACCTTCTGGGTTTGTTACTAGACTCCAATATGAAGGAGGGTGAAGAGCATGGCAAG
TCCCAAAACAAAGGGATGAGCACTGAGGAAGTGATTGAAGAGTGCAAGCTGTTCTACTTT
GCAGGGCAAGAGACTACCCCCTCACTTTTACTCACATGGACAATGATTTTATTGAGCATGTA
TCCGAACTGGCAGACTAAGGCAAGAGAAGAGGTTCTTAAAGTCTTTGGAAAGAACACACC
AGACATGGAGGGATTGAGCCACTTGAAGATTGTGACCATGATTCTATATGAAGTTCTTAGGT
TATATCCACCAGTGGTTTTCATAACAAGAAAAACCTACAAAGCAATGGAACTCGGTGGGAT
TACTTACCCTCCAGGAGTGATATTCTCATTGCCTTTACTCTTCATTCACCATGACCCTACTTTT
TGGGGAGAAGATGCCAAAGAGTTTAATCCAGAGAGGTTTTCCGAAGGGATATCGAAAGCAT
CCAAAGTTCCGGCTGCCTTCTTTCCTTTCGGTGGAGGTCCACGCATTTGCATTGGTCAAAAC
TTTGCACTGATTGAAGCTAAGATAGGAATTTGCATGATTCTTCAGCACTTTTCCTTTGTACTT
TCGCTGCTTTCCTATATTCATGCACCGCACACTGTTATTACTCTTCAACCAGAGCATGGAGCT
CAACTCATGCTGCAAAAGTTGTGA

>DzCYP72A5

ATGGAGTCAGTGATGGGAGTGGTATGGGCGGCGGCGGTGGCGGTGGTGGCGTGGGCG
TGGAGGACGTTGGATTGGGTTTGGTGGACGCCGAGGAGGCTGGATCGGGAGCTCCGGCAG
CAGGGCCTGCGCGGCAACCAGTACCGAGTCTTGCACGGCGATCTCAAGGAAAACACCCGG
CTATCCGAGGAGGCTAAATCCCGGCCTCTGCCTCTTCACTGCCATGACATCGCCCCCCGCGT
TGTCCCTGTCCTCCACAACGCCATCAAAGATCACGGTAAAATCTCAATAACTTGGTTTGGGC
CTTACCCAAGAGTGACCTTAATGGAGCCAGAGCTAGTGAAAGAAGTGTTGTCAAACAAGT
TTGGACATTTTGCTAAAGTAAGACCAAATCCCCTTACCAAATTATTGGTCCAAGGACTTGTA
GTTTATGAAGGTGAAAAGTGGGCCAAACACAGAAGGATCATCAACCCTGCATTCCATCTTG
AGAAACTAAAGCTAATGCTTCCAGCATTCTCTACATCTTGTGGTGAACTGATTAGAAGATGG
GAGAAGATGATCCCTGATGAAGGCTCCCATGAACTAAATGTCTTTCCAGAGCTCCAAGACC
TCACAAAGATGTCATCTCCAGGACTGCATTCGGTAGCAGCTATGAAGAAGGAAGGAAGAA
GAATATTTGAACTACTAGCAGAGCAAATTCAGCTTCTTATCCCAGCTTTCCAGACTTTATACA
TCCCTGGTTATCGATTTCTGCCCACACCAATGAACAAAAGAAGAAGCCAAGTGTACAATGA
GATGAAAAGAATTCTTAAAGGCATGATCGAGAAGAGAGAGAAGGCCATAAGAATGGGGGA
AGGTAGCAAGAATGACCTTCTGGGTTTGTTACTAGACTCCAATATCAAAGAGAGTGAAGAG
CATGGGAAGTCCCAAAACAAAGTGATGAGCACTGAGGATGTGATTGAAGAGTGCAAGCTA
TTCTACTTTGCAGGGCAAGAGACTACATCACTTTTACTCACATGGACAATGATTTTATTGAGT
ATGTATCCGAACTGGCAGGCTAAGGCAAGAGAAGAGGTTCTTCAAGTCTTTGGAAAGAAC
ACACCAGACATGGAGGGATTGAGCCACTTGAAGATTGTGACCATGATTCTATATGAAGTTCT
GAGGTTATATCCACCATTTGTTTTACTAAGAAGAAAAACCTACAAAGCAATGGAACTCGGT
GGGATTACTTACCCTCCGGGAGTGATACTCTCACTGCCTCTACTCTTCATTCACCATGACCCT
GCTTTTTGGGGAGAAGACGCCAAAGAGTTTAATCCAGAGAGGTTTTCCGAAGGGATATCGA

AAGCATCCAAAGTTCCAGGTGCCTTCTTTCCTTTCGGTGGAGGTCCACGCATTTGCATTGGC
CAAAGCTTTGCAATGATTGAAGCTAAGATAGGAATTTGCATGATTCTTCAGTGTTTCTCCTTT
GAGCTTTCACCTTCCTATATCCATGCACCGCACACTGTTATTACTCTTCAACCACAGCATGGG
GCTCAACTCATGCTGCAAAAGCTGTGA

>DzCYP72A6

ATGGAGTTAGTGATGGGAGTGGTATGGGCGGCGGTGGCGGTGGTGGCGTGGGCGTGGAGG
ACGTTGGATTGGGTTTGGTGGACGCCGAGGAGGCTGGACCGGGAGCTCCGGCGGCAGGGC
CTGCGCGGCAACCAGTACCGAGTATTGCACGGCGATCTCAAGGAAAACGCCCAGCTCTCCG
AGGAGGCTAAATCCCGGCCTCTGCCTCTTCACTACCATGACATCGCCCCCCGCGTTCTCCCT
CTCTTCCACAACGCCATCAAAGATCACGAGCCAGAGCTAGTGAAAGAAGTACTCTCAAAC
AAGTTTGGACATTTTGTTAAACCAAGTACAACTCCCCTTGCCAAATTTTTGGTCCAAGGGCT
TGCGTCTTATGACGGTGAAAAGTGGGTCAAACACAGAAGGATCATCAACCCTGCATTCCAT
TTTGAGAAACTAAAGCAAATGCTGCCAGCATTCTCTACGTCTTGTGGTGAACTTATTAGAAG
ATGGAACAAGATGATCCCTGATGAAGGCTCCCAAGAACTAAATGTCTTTCCAGAGCTCCAA
GGCCTCACAAAAGATATCATCTCCAGAACCGCATTCGGTAGCAGCTATGAAGAAGGGAGAA
GAATATTTGAACTCCTAACTGAACAAATTAAGCTTACTATTCCAGCTTTCAAGACTGTATACA
TCCCTGGTTATCGATTTCTGCCCACACCAATGAACAAAAGAAGAAGCCAAGTGTACAATGA
GATGAAAAGAATTCTTAAAGGCATGATTGAGAAGAGAGAGAAGGCCATAAGAATGGGGGA
AAGTAGCAAGAATGACCTTCTGGGTTTGCTACTAGACTCCAATATGCAGGAGGGTGAAGAG
CATGTGAAGTCCCAAGACAAAGGGATGAGCACTGAGGATGTGATTGAAGAGTGCAAGCTG
TTTTACATTGCAGGGCAAGAGACTACATCAGCTCTACTCACTTGGACAATGATTTTATTGAG
CATGTATCCGAACTGGCAGGCTAATGCAAGAGAAGAGGTTCTTCAAGTCTTTGGAAAGAGC
ACACCAGACATAGAGGGATTGAGTCACTTGAAGATTGTGACCATGATTCTATATGAAGTTCT
CAGGTTATATCCACCAGGGGTTTTTCTTGATAGCAAAACCTCCAAAGCAATGGAACTTGGTG
GGATTACTTACCCTTCAGGAGTGATACTCTCACTACCTCTACTCTTCATTCACAATGACCCTA
CTTTCTGGGGAGAGGATGCCAAAGAGTTCAATCCAGAGAGGTTTTCCGAAGGGATATCGAA
AGCATCCAAAGTTACAGGGGCCTTCTTTCCTTTTGGTGGAGGTCCGCGCATTTGCATTGGCC
AAAACTTTGCACTGATGGAAGCTAAGATAGGAATTTGCATGATTATTCAGCACTTCTCATTT
GTGCTTTCACCTTCCTATATCCATGCACCGCACTCTGTTATTACTCTTCAACCACAGCATGGA
GCTCAACTCATGCTGCAAAAGCTGTGA

>DzCYP72A7

ATGAGGCTGGACCGGGAGCTCCGGCGCCAGGGCCTGCGCGGCAACCAGTACCGAGTCTTC
CATGGCGATCTCAAGGAAAACGCCCGGCTCTCGAAGGAGGCTGAATCCCGGCCTCTGCCTC
TTCACTGCCATGACATCGCCCCCCGCGTTCTCCCTCTCTTCCACAACGCCATCAAAGATCAC
GGTAAAATCTCAATAACTTGGCTTGGCCCTTGTCCAAGAGTGACCTTAACGGAACCAGAAC
TAGTAAAAGAAGTGTTAAACAAGTTTGGACATTTTGTTAAACCAAGTACAACTCCTTTTGCC
AAATTTTTGGTCCAAGGGCTTGTGTCTTATGAAGGTGAAAAGTGGGCCAAACATAGAAGGA
TCCTCAACCCTGCATTCCATCTAGAGAAACTAAATCTAATGTTGCCAGCATTCTCTACATCTT
GTAGTGAATTGATTAGAAGATGGGAGAAGATGATTCCTGATGAAGGCTCCCAAGAACTAAA
TGTCTTTCCAGAGCTCCAAGGCCTCACAAAAGATGTCATCTCTAGGACTGCATTCAGTAGC
AGCTATGAAGAAGGGAGAAGAATATTTGAACTCCTAAAAGAGCAAATTCAGCTTTATATTCA
AGTTTACAAGACTGTATACATCCCTGGTTATCGATTTCTGCCCACACCAATGAACAAAAGAA
GAAGCCAAGTATACAATGAGATGAAAAGAATTCTTAAAGGCATGATTGAGAAGAGAGAAA
AGGCCATAAGAATGGGGGAAAGTTGTAAGGATGACCTTCTGGGTTTGTTACTAGACTCCAA

TATGAAGGAGGGTGAAGAGCATGGCAAGTCCCAAAACAAAGGGATGAGCACTGAGGAAG
TGATTGAAGAGTGCAAGCTGTTCTACTTTGCAGGGCAAGAGACTACCTCACTTTTACTCAC
ATGGACAATGATTTTATTGAGCATGTATCCGAACTGGCAGACTAAGGCAAGAGAAGAGGTT
CTTAAAGTCTTTGGAAAGAACACACCAGACATGGAGGGATTGAGCCACTTGAAGATTGTGA
CCATGATTCTATATGAAGTTCTTAGGTTATATCCACCAGTGGTTTTCATAACAAGAAAAACCT
ACAAAGCAATGGAACTCGGTGGGATTACTTACCCTCCAGGAGTGATATTCTCATTGCCTTTA
CTCTTCATTCACCATGACCCTACTTTTTGGGGAGAAGATGCCAAAGAGTTTAATCCAGAGAG
GTTTTCCGAAGGGATATCGAAAGCATCCAAAGTTCCGGCTGCCTTCTTTCCTTTCGGTGGAG
GTCCACGCATTTGCATTGGTCAAAACTTTGCACTGATTGAAGCTAAGATAGGAATTTGCATG
ATTCTTCAGCACTTTTCCTTTGTACTTTCGCTTTCCTATATTCATGCACCGCACACTGTTATTA
CTCTTCAACCAGAGCATGGAGCTCAACTCATGCTGCAAAAGTTGTGA

>DzCYP72A8

ATGGAGTCAGTGATGGGAGTGGTATGGGCGGCGGCGGCGGTGGCGGTGGTGGCGTGGGCG
TGGAGGACGTTGGATTGGGTTTGGTGGAAGCCGAGGAGGCTGGATCGGGAACTCCGGCGG
CAGGGCCTGCGCGGCAACCAGTACCGACTCTTGCACGGCGATCTCAAGGAAAACGCCCGG
CTCTCCGAGGAGGCTAAATCCCGGCCTCTGCCTCTTCACTGCCATGACATCGCCCCCCGCGT
TCTCCCTGTCCTCCACAACGCCATCAAAGATCACGGTAAAATCTCAATAACTTGGTTTGGGC
CTTACCCAAGAGTGACCTTAATGGAGCCAGAGCTAGTCAAAGAAGTGTTGTCAAACAAATT
TGGACATTTTGCTAAAATAAGAGCAACTCCCCTTGCCAATTTTTTGGTCCAAGGACTTGTGA
CTTATGAAGGTGAAAAGTGGGCCAAACATAGAAGGATCATCAACCCTGCATTCCATCTTGA
GAAACTAAAGCTAATGCTTCCAGCATTCTCTACATCTTGTGGTGAACTGATTAGAAGATGGG
AGAAGATGATCCCTGATGAAGGCTCCCAAGAACTAAATGTCTTTCCAGAGCTCCAAGACCT
CACAAAAGATGTCATCTCCAGGACTGCATTCGGTAGCAGCTATGAAGAAGGAAGAAGAATA
TTTGAACTCCTAGCAGAGCAAATTCAGCTTCTTATCCCAGCTTTCCAGACTATATACATCCCT
GGTTATCGATTTCTGCCCACACCAATGAACAAAAGAAGAAGCCAAGTGTACAATGAGATGA
AAAGAATTCTTAAAGGCATGATCGAGAAGAGAGAGAAGGCCATAAGAATGGGGGAAGGTA
GCAAGAATGACCTTCTGGGTTTGTTACTAGACTCCAATATCAAAGAGAGTGAAGAGCATGG
GAAGTCCCAAAACAAAGTGATGAGCACTGAGGATGTGATTGAAGAGTGCAAGCTATTCTAC
TTTGCAGGGCAAGAGACTACATCACTTTTACTCACATGGACAATGATTTTATTGAGCATGTAT
CCAAACTGGCAGGGTAAGGCAAGAGAAGAGGTACTTCAAGTCTTTGGAAAGAGCACACCA
GATATGGAGGGATTGAACCACTTGAAGATTGTGACCATGATTCTATATGAAGTTCTGAGGTT
ATATCCACCATTTGTTTTCCTAACAAGAAAAACCTACAAAGCAATGGAACTCGGTGGGATTA
CTTACCCTCAAGGAGTGATACTCTCACTGCCTCTACTCTTCATTCACCATGACCCTGCTTTTT
GGGGAGAAGACGCCAAAGAGTTTAATCCAGAGAGGTTTTCCGAAGGGATATCAAAAGCAT
CCAAAGTTCCGGGTGCCTTCTTTCCTTTCGGTGGAGGTCCGCGCATTTGCATTGGCCAAAG
CTTTGCAATGATTGAAGCTAAGATAGGAATTTGCATGATGCTTCAGCGCTTCTCCTTTGAGC
TTTCGCCTTCCTATATCCATGCACCGCACACTGTTATTACTCTTCAACCACAGCATGGGGCTC
AACTCATGCTGCAAAAGCTGTGA

>DzCYP72A9

ATGGAGTCAGTGATGGGAGTGGTATGGGCGGCGGCGGCGGTGGCGGTGGCGGTGGTGGCG
TGGGCGTGGAGGACGTTGGATTGGGTTTGGTGGACGCCGAGGAGGCTGGATCGGGAGCTC
CGGCAGCAGGGCCTGCGCGGCAACCAGTACCGAGTCTTGCACGGCGATCTCAAGGAAAAC
GCCCGGCTATCCGAGGAGGCTAAATCCCGGCCTCTGCCTCTTCACTGCCATGACATCGCCCC
CCGCGTTGTCCCTGTCCTCCACAACGCCATCAAAGATCACGGTAAAATCTCAATAACTTGGT

TTGGGCCTTACCCAAGAGTGACCTTAATGGAGCCAGAGCTAGTGAAAGAAGTGTTGTCAA
ACAAGTTTGGACATTTTGCTAAAGTAAGACCAAATCCCCTTACCAAATTATTGGTCCAAGGA
CTTGTAGTTTATGAAGGTGAAAAGTGGGCCAAACACAGAAGGATCATCAACCCTGCATTCC
ATCTTGAGAAACTAAAGCTAATGCTTCCAGCATTCTCTACATCTTGTGGTGAACTGATTAGA
AGATGGGAGAAGATGATCCCTGATGAAGGCTCCCATGAACTAAATGTCTTTCCAGAGCTCC
AAGACCTCACAAAAGATGTCATCTCCAGGACTGCATTCGGTAGCAGCTATGAAGAAGGAAG
AAGAATATTTGAACTCCTAGCAGAGCAAATTCAGCTTCTTATCCCAGCTTTCCAGACTATATA
CATCCCTGGTTATCGATTTCTGCCCACACCAATGAACAAAAGAAGAAGCCAAGTGTACAAT
GAGATGAAAAGAATTCTTAAAGGCATGATAGAGAAGAGAGAGAAGGCCATAAGAATGGGG
GACAGTAGCAAGAATGACCTTCTGGGTTTGTTACTAGACTCCAATATGAAGGAGGGTGAAG
AGCATGGGAAGTCCCAAAACAAAGGGATGAGCACTGAGGATGTGATTGAAGAGTGCAAGC
TGTTCTACTTTGCAGGGCAAGAGACTACAGCAGTTCTACTCACATGGACAATGATTTTATTG
AGCATGTATCCAAACTGGCAGGGTAAGGCAAGAGAAGAGGTACTTCAAGTCTTTGGAAAG
AGCACACCAGATATGGAGGGATTGAACCACTTGAAGATTGTGACCATGATTCTATATGAAGT
TCTGAGGTTATATCCACCATTTGTTTTCCTAACAAGAAAAACCTACAAAGCAATGGAACTCG
GTGGGATTACTTACCCTCAAGGAGTGATACTCTCACTGCCTCTACTCTTCATTCACCATGACC
CTGCTTTTTGGGGAGAAGACGCCAAAGAGTTTAATCCAGAGAGGTTTTCCGAAGGGATATC
GAAAGCATCCAAAGTTCCGGGTGCCTTCTTTCCTTTCGGTGGAGGTCCGCGCATTTGCATTG
GCCAAAGCTTTGCAATGATTGAAGCTAAGATAGGAATTTGCATGATTCTTCAGCGCTTCTCC
TTTGAGCTTTAA

>DzCYP72A10

ATGGAGTTAGTGATGAGAGTGGTATGGGCGGTGGCGGCGGTGGCGGTGGTGGCGTGGGCG
TGGAGGACGTTGGATTGGGTTTGGTGGACGCCGAGGAGGCTGGATCGGGAGCTCCGGCGG
CAGGGCCTGCGCGGCAACCAGTACCGACTCTTGCACGGCGATCTCAAGGAAAACGCCCAG
CTCTCCGAGGAGGCTAAATCCCGGCCTCTGCCTCTTCACTACCATGACATCGCCCCCCGCGT
TCTCCCTCTCTTCCACAACGCCATCAAAGATCACGGTAAAATCTCAATAACTTGGCTTGGTC
CTTACCCAAGAGTGATCTTAACAGAGCCAGAGCTAGTGAAAGAAGTACTCTCAAACAAGTT
TGGACATTTTGTTAAACCAAGTACAACTCCCCTTGCCAAATTTTTGGTCCAAGGGCTTGCGT
CTTATGACGGTGAAAAGTGGGTCAAACACAGGAGGATCATCAACCCTGCATTCCATTTTGA
GAAACTAAAGCAAATGCTGCCAGCATTCTCTACGTCTTGTGGTGAACTTATTAGAAGATGGA
ACAAGATGATCCCTGATGAAGGCTCCCAAGAACTAAATGTCTTTCCAGAGCTCCAAGGCCT
CACAAAAGATATCATCTCCAGAACCGCATTCGGTAGCAGCTATGAAGAAGGGAGAAGAATA
TTTGAACTCCTAACTGAACAAATTAAGCTTACTATTCCAGCTTTCAAGACTGTATACATCCCT
GGTTATCAATTTCTGCCCACACCAATGAACAAAAGAAGAAGCCAAGTGTACAATGAGATGA
AAAGAATTCTTAAAGGCATGATTGAGAAGAGAGAGAAGGCCATAAGAATGGGGGAAAGTA
GCAAGAATGACCTTCTGGGTTTGCTACTAGACTCCAATATGCAGGAGGGTGAAGAGCATGG
GAAGTCCCAAGACAAAGGGATGAGCACTGAGGATGTGATTGAAGAGTGCAAGCTGTTTTA
CATTGCAGGGCAAGAGACTACATCAGCTCTACTCACTTGGACAATGATTTTATTGAGCATGT
ATCCGAACTGGCAGGCTAATGCAAGAGAAGAGGTTCTTCAAGTCTTTGGAAAGAGCACAC
CAGACATAGAGGGATTGAGCCACTTGAAGATTGTGACCATGATTCTATATGAAGTTCTCAGG
TTATATCCACCAGGGGTTTTTCTTGATAGAAAAACCTACAAAGCAATGGAACTTGGTGGGAT
TACTTACCCTTCAGGAGTGATACTCTCACTACCTCTACTCTTCATTCACAATGACCCTACTTT
CTGGGGAGAGGATGCCAAAGAGTTCAATCCAGAGAGGTTTTCCGAAGGGATATCGAAAGC
ATCCAAAGTTCCAGGGTCCTTCTTACCTTATGGTGTAGGTCCGCGCATTTGCATTGGCCAAA

ACTTTGCACTGATGGAAGCTAAGATAGGAATTTGCATGATTATTCAGCACTTCTCATTTGTGC
TTTCACCTTCCTATATCCATGCACCGCACTCTGTTATTACTCTTCAACCACAGCATGGAGCTC
AACTCATGCTGCAAAAGCTGTGA

>DzCYP72A11

ATGGAGTTAGTGATGGGAGTGGTATGGGCGGCGGCGGTGGTGATGCTGGTGGCGTGGGCGT
GGAGGACGTTGGATTGGGTTTGGCGGACGCCGATGAGGCTGGACCGGGAGCTCCGGCGCC
AGGGCCTGCGCGGCAACCAGTACCGAGTCTTCCATGGCGATCTCAAGGAAAACGCCCGGC
TCTCGAAGGAGGCTGAATCCCGGCCTCTGCCTCTTCACTGCCATGACATCGCCCCCCGCGTT
CTCCCTCTCTTCCACAACGCCATCAAAGATCATGGTAAAATCTCAATAACTTGGCTTGGCCC
TTATCCAAGAGTGACCTTAACGGAACCAGAACTAGTTATAGAAGTGTTAAACAAGTTTGGA
CATTTTGTTAAACCAAGTACAACTCCTTTTGCCAAATTTTTGGTCCAAGGGCTTGTGTCTTAT
GAAGGTGAAAAGTGGGCCAAACATAGAAGGATCCTCAACCCTGCATTCCATCTTGAGAAA
CTAAAGCTAATGTTGCCAGCATTCTCTACATCTTGTAGTGAATTGATTAGAAGATGGGAGGA
GAAGATTCCTGATGAAGGCTCCCAAGAACTAAATGTCTTTCCAGAGCTCCAAGGCCTCACA
AAAGATGTCATCTCTAGGACTGCATTCAGTAGCAGCTATGAAGAAGGGAGAAGAATATTTG
AACTCCTAAAAGAGCAAATTCAGCTTTATATTCAAGTTTACAAGACTGTATACATCCCTGGTT
ATCGATTTCTGCCCACACCAATGAACAAAAGAAGAAGCCAAGTATATAATGAGATGAAAAG
AATTCTTAAAGGCATGATTGAGAAGAGAGAAAAGGCTATAAGAATGGGGGAAAGTTGTAA
GGATGACCTTCTGGGTTTGTTACTAGACTCCAATATGAAGGAGGGTGAAGAGCATGGCAAG
ACCCAAAACAAAGGGATGAGCACTGAGGAAGTGATTGAAGAGTGCAAGCTGTTCTACTTT
GCAGGGCAAGAGACTACCTCACTTTTACTCACATGGACAATGATTTTATTGAGCATGTATCC
GAACTGGCAGGCTAAGGCAAGAGAAGAGGTTCTTCAAGTCTTTGGAAAGAACACACCAGA
CATGGAGGGATTGAGCCACTTGAAGATTGTGACCATGATTCTATATGAAGTTCTTAGGTTATA
TCCACCAGTGGTTTTCCTAACAAGAAAAACCTACAAAGCAATGGAACTCGGTGGGATTACT
TACCCTCCAGGAGTGATATTCTCATTGCCTTTACTCTTCATTCACCATGACCCTACTTTTTGG
GGAGAAGATGCCAAAGAGTTTAATCCAGAGAGGTTTTCTGAAGGGATATCGAAAGCATCCA
AAGTTTCGGCTGCCTTCTTTCCTTTCGGTGGAGGTCCACGCATTTGCATTGGTCAAAACTTT
GCACTGATTCAAGCTAAGATAGGAATCTGCATGATTCTTCAGCACTTTTCCTTTGTGCTTTCG
CTTTCCTATATTCATGCACCGCACACTGTTATTACTCTTCAACCACAGCATGGAGCTCAACTC
ATGCTGCAAAAGTTGTGA

>DzCYP72A12

ATGGAGTCACTGATGGGAGTGATATGGGCGGTGGCGGCGGTGGTGGTGGTGGCGGCCGCG
TGGAGGACGTTGGATTGGATTTGGTGGACGCCGAGGAGGCTGGACCGGGAGCTCCGACGC
CAGGGCCTGCGCGGCAACCAGTACCGAGTCTTGCACGGCGATCTCAAGGAAAACGTCCGG
CTCTCGAAGGAGGCTAAATCCCGGCCTCTGCCTCTTCACTGCCATGACATCGCCCCCCGCGT
TCTCCCTCTCTTCCACAACGCCATCAAAGATCACGGTAAAATCTCAATAACTTGGCTTGGCC
CTTATCCAAGAGTGACCTTAACAGAGCCAGAGCTAGTGAAAGAAGTGCTGTCAAACAAGT
TTGGACATTTTGTTAAACCAACTACAACTCCCTTAGCCAAATTTTTGGTCCAAGGGCTTGTG
TCTTATGAAGGTGAAAAGTGGGCCAAACACAGAAGGATCATCAATCCTGCATTCCATCTTG
AGAAACTAAAGCTAATGCTGCCAGCATTCTCTACATCTTCTGGTGAACTGATTAGAAGATGG
GAGAAGATGATTCCTAATGAAGGCTCCCAAGAACTAAATGTCTTTCCAGAGCTCCAAGACC
TCACAAAAGATGTCATCTCCAGGACTGCATTCGGTAGCAGTTACGAAGACGGAAGAAGAAT
ATTTGAACTCCTAACAGAGCAAATTCAGCTTCTTATTCCAGCTTTCCAGACTGTATACATTCC
TGGTTTTCGATTTCTGCCCACACCAATGAACAAAAGAAGCAGCCAAGTGTACAATGAGATG

AAAAGAATTCTTATAGGCATGATTGAGAAGAGAGAGAAGGCCATAAGAATGGGGGAAAGT
AACAAGAATGACCTTCTGGGTTTGTTACTAGACTCCAATATGAAGGAGGGTGAAGAGCATA
GGAAGTCCCAAAACAAAGGGATGAGTACTGAGGATGTGATTGAAGAGTGCAAGCTGTTCT
ACTTTGCAGGGCAAGAGACTACATCAGTTCTACTCACATGGACAATGATTTTATTGAGCATG
TATCCAAATTGGCAGGCTAAGGCAAGAGAAGAGGTTCTTCAAGTCTTTGGAAAGAGCGCA
CCAGATATGGAGGGATTGAGCCACTTGAAGATTGTGACCATGATTCTATATGAAGTTCTAAG
GTTATATCCACCAGCGGTTTTCCTAACAAGAAAAACCTACAAAGCAATGGAACTCGGTGGG
ATTACTTACCCTCCAGGAGTGATACTCTCACTGCCTCTACTCTTCATTCACCATGACCCTGTT
TTCTGGGGAGAAGACGCCAAAGAGTTTAATCCAGAGAGGTTTTCCGAAGGGATATCGAAA
GCATCCAAAGTTCCAGGTGCCTTCTTTCCTTTCGGTGGAGGTCCGCGCATTTGCATTGGCCA
AAACTTTGCACTGATTGAAGCTAAGATAGGAATTTGCATGATTCTTCAGCACTTCTCCTTTG
TGCTTTCGCCTTCCTATATCCATGCACCGCACAATGTTATTACTCTTCAACCACAGCATGGAG
CTCAACTCATGCTGCAAAAGCTGTGA

>DzCYP72A13

ATGGAGTTAGTGATGGGAGTGATATGGACGGTGACGGCGGCGGTGGTGGTGGTGTGGGCGT
GGAGGACGTTGGATTGGGTTTGGTGGACGCCGAGGAGGCTGGACCGGGATCTCCGGCGCC
AGGGCCTGCGCGGCAACCAGTACCGATTATTGCACGGCGATCTCAAGGAAAACGCCCGGCT
CTCCAAGGAGGCTACATCCCGGCCTTTGCCTCTTCACTGCCATGACATCGCTCCCAGGGTTG
CCCCTCTCATCCACAACGCCATCAAAGATCACGGTAAAATATCAATAACTTGGCTTGGACCT
TATCCAAGAGTGTCCTTGATGGACCCAGATTTAGTGAAAGAAGTACTGTCAAACAAGTTTG
GACATTTTGTTAAACCAAGAATAACTCCTATTGCCAAATTTTTGGTCCAAGGACTTGTAGCT
TATGAAGGTGAAAAGTGGGCCAAACACAGAAGGATAATTAACCCCGCATTCCATCTTGAGA
AACTAAAGCTAATGCTGCCAGCATTCTCTACATCTTCTGGTGAACTGATTAGAAGATGGGAG
AAGATGATCCCTGATGAAGGCTCCCAAGAACTAAATGTCTCGCCAGAGATCCAAAACCTCA
CAGGAGATGTCATCTCCAGGACTGCATTCGGTAGCAGCTATGAAGAAGGGAGAAGAATATT
TGAACTCCTAACAGAGCAAATTCATCTTACTATTCCAGCTTTGCAGACTGTATACATCCCTGG
TTATCGGTTTCTGCCCACACCAATGAACAACAGAAGAAGCCAAGTTTACAATGAGATGAAA
AGAATTCTTAAAGGCATGATTGAGAAGAGAGAGAAGGCCATAAGAATGGGGGAAAGTAGC
AAGAATGACCTTCTGGGTTTGTTACTAGACTCCAATATGAAGGAGGGTGAAGAGCAGGAGA
AGTCCCAAAACAAAGTGATGAGCACTGAGGATGTGATTGAAGAGTGCAAGCTGTTCTACTT
TGCAGGGCAAGAGACTACATCAGTTCTACTCACATGGACAATGATTTTATTGAGCATGTATC
CTAACTGGCAGGCCAAGGCAAGAGAAGAGGTTCTTCAAGTCTTTGGAAAGAGCACACCAG
ACATGGATGGATTGAGCCGCTTGAAGATTGTGACCATGATTCTGTATGAAGTTCTGAGGTTA
TATCCACCGGTGACTTTCCTAACCAGAAAAACATACAAAGCAATGGAACTTGGTGGGATCA
ATTACCCTCCAGGAGTGTTACTCCAACTGCCTCTACTCTTCATTCACCATGACCCTGAATTCT
GGGGAGAAGACGCCAAAGAGTTCAATCCCGAGAGGTTTTCTGAAGGGATATTGAAAGCAT
CCAAAGTTCCGGGTGCCTTCTTTCCTTTCGGTGGAGGTCCGCGCATTTGCATTGGCCAAAG
CTTTGCACTGATTGAAGCTAAGATTGGAATTAGCATGATTCTTCAGCACTTCTCCTTCGTGCT
CTCGCCTTCGTATATCCATGCACCGCACACTGCTCTTACTCTTCAACCACAGCATGGAGCTC
AACTCATGCTTCAAAAACTCTGA

>DzCYP72A14

ATGGAGTACTCAGTGGCGGCAGGGGTGAAGGAGATGATATGGGGTGTGGCAACGGCGCTG
TTGGTGGTGTGGGTGTGGAGGACGTTGGAGTGGTTGTGGTGGAAGCCGAGGAGGCTGGAG
CGCGAGCTGAAGAGGCAGGGATTGCGCGGAGGCAAGTACCGTCTCTTCCACGGCGATCTC

AAGTACAACGCGCGACTCATGAAGGATGCTCTTTCTCGCCCCTTGCCCCCTTACTCTCACGA
CGTCGCCCCACGAGTCATCCCTCTCGTCCATCAAGCTATCAAGATTCATGGTAAAATGTCTAT
AACATGGCTTGGGCCTTATCCTAGAGTGAGCTTGATGGACCCAGAGTTAATAAGAGAAGTT
CTATCAAACAAGTTTGGCCACTTTGTGAAGCCAAACTTCAGTCCCCTTGTGAAACTGTTAG
CCCAAGGGCTTGCAAGTCATGAAGGTGAGAAGTGGGCTAAACATAGGAGGATCATCAACC
CTGCTTTCCATTTTGAAAAACTAAAGTGCATGTTGCCGGCTTTTTCGACATGTTGTGATGAA
CTAGTTAAGAGATGGCAGAACAAGATGAATGTAGAGGGTTCCATTGAGCTGAATATGTGGC
CAGAGCTCCAAAATCTTACTGGAGATGTCATCTCCAGGACTGCATTTGGTAGTTGCTACGAA
GAAGGAAGAAGAATTTTTCAACTCCAAACAGAGCAAGCTGAACTTATTATTCCAACCGCTC
AGACTGTATATGTTCCTGGTTTCAGGTTTTTACCAACACCAAAGAACAACAGAAGAAAAGC
AATTGATAGAGAGATTAAAACAATTCTGAGAAGCATGATTGAAAAAAGAGAGAAGGCCATA
AGAATGGGAGGAGAAACCAGCTGCAAGGATGACCTTCTGGGTTTGCTATTGGAGTCCAAC
ATGAAGGAGACTGAACAACAAGGCAGGTCCAAAAACAAAGGGCTGACTACTGAGGACGT
GATTGAAGAATGCAAGCTCTTCTACTTAGCAGGGCAAGAGACCACATCATCTCTTCTCACAT
GGACAATGGTTTTGTTGAGCATGTATCCAAACTGGCAGGCTAAAGCCAGAGAAGAAGTTCT
CCAAGTCTTCGGGAAAAACAAACCCGACATGGACGGCGTCGGCCGCCTGAAGATCGTGAC
GATGATACTGTATGAGGTTCTCAGATTATATCCACCAGCCATTTCACTGACAAGAAGAACAT
ACAAAACAATGGAACTGGGAGGGATAACTTATCCTCCAGGGGTGCTGCTCTTGTTACCCAT
AATCTTGGTTCACCATGACTTGGATTTCTGGGGAGAAGATGCCAAAGAGTTTAAGCCGGAT
AGGTTTGCAGAAGGGATATCAAAGGCATCCAAAGTTCCAGGAGCTTTCTTTCCTTTTGGTG
GAGGTCCACGTATATGCATTGGCCAAAGCTTTGCACTCATTGAAGCTAAGCTGGGACTTAGC
ATGATTCTTCAGAACTTCTCCTTTGAGCTCTCACCTTCTTATATCCATGCTCCTTACACTCTTG
TTACTCTTCAGCCTCAGCATGGAGCTCCAATCAAGCTGCACAAACTCTGA

>DzCYP72A15

ATGGAGCCGAGCTTCATCTCCATGGCAGCAACCCTATCATCCCTTCTGCTTCTCTACTGTGCT
CTAACGGTGGTCCATGTTGTATGGTGGAGGCCAAGGATGATAGAGAAGCAGCTCAAGAAGC
AAGGGATCAAGGGGAGGCCATACAAGGTTTTGCGTGGGGATCTCAGTGACATAGTCAAGAT
CATGAAGGAGGCTTTGTCCAAACCCATGGAGCTTCATCACCATATCAGCCCCCGTGCTCTCC
CCTTTGTCCACAGTACTGTGGAGCAATTTGGCAAATTATCAATAATTTGGTATGGGAAAAAT
CCAAGGATAATCATACAAGATGTAGAACTGATAAAGGAAGTACTGGCAAACAAAAATGGGA
ATTTTCTGAAGCCACTTCTGAATCCGCTCCAAAGACTATTAGCTGAGGGTGTTTCCCTTGTT
GAGGGAGATAAATGGGTTCAACATAGAAACATACTAAATCCTGCATTCCATTTGGCCAAGTT
GAAGGGGATGGTACCAGCATTTTGTACTAGTTGCAGTGAAATGATCAGTAAATGGGAAATG
TTGTTTGGTCCTGAAGGATCTTGTGAACTGGATGTTTGGATAGAGCTGAAAGCCCTAACAG
CCGATGTTATATCTCGCACGGCATTTGGTAGCAACTACAGAGAAGGGCAGAAGGTGTTCGA
GTTTCAGCAGGAACAGATTCAACTCATGATGGAAGCCTCTTGGATTCCGTATATACCAGGAT
TCAGGTTTTTGCCTACAAAGAAGAACCGGAGGAGGTACTATTTGGACAATGAGATTAAGGC
AATAATAAGATCTTTGATTCACAAGAAAGAGAAGAGCATGGAAGTTGGTGAATCTGGTGGT
GAAGATTTATTGTCCTTGTTGCTGCAATCAAATCATAATGTGCATGAAAATGCTGCAAATGGT
TTGAAGCTTGAAGGATTGACAATTGATGAAGTGATTGAGGAATGCAAGCTCTTCTACTTTGC
TGGTCATGATACGACTTCTAGTTTGCTGACCTGGACACTGATACTTTTATCAATGTATCCTGC
TTGGCAAACTAGAGCAAGAGAAGAGGTGCATCGAATTTGTGGGAAGAACATGCCAGACTA
TGAAAGCATTGGCCAATTTAAGATTGTGACGATGATATTGCACGAAGTTCTAAGATTATATCC
ACCGGTAACAGGCCAGTATCGGCATGTGTACCACGAAACGAAGCTAGGGGAATTATCTCTT

CCTGCTGGTGTGGATCTTTTGTTCCTTCCCTTCTTGTCTACCATGATCCTGAGATTTGGGGT
GAAGACTGCAAAGAATTCAATCCCCAGAGGTTCTCCGAGGGCGTGCTCAAGGCGACGAAG
AATCAGTTTGTGTATTTCCCTTTTGGTTTTGGCCAAAGAACTTGCATTGGCCAGAACTTCGC
CATGATTGAAGCGAAGATTGCTCTTGCAATGATGCTGCAGCATTTCTCATTTGATCTTTCACC
ATCCTATGCGCACTGCCCTTACAGTCTCATTACACTTCAACCACAACATGGAGCTCAGCTCA
TTGTACATCGTCTTTGA

>DzCYP72A16

ATGGTGGTGGTGGTGGTGTTTGTGAGTTTAGTGGTGATTGTGGGATTGGTGGCTAGAGTTTC
ATGGTGGATATGGTGGAAGCCAAAGATGATGGAGAAGGAGATGAGGAGGCAGGGGATTAA
TGGAACCAAATATAGGACATTTATGGGGGATCTCAAGGATGAGGGGGAGGCTTACAAGGAG
GCTTGGTCCAAACCTATGGATCTCAACCACAACATTGTCCCACGTGTGCTCCCACATGCTCA
TCTCATGCTTCAAACTTATGGAAAGACATCATTCAAATGGATGGGAACAACTCCAGCAGTG
ACTATATGGGAGCCAGAATTGTTAAGAGAGGTCCTAATGAACAAATGTGGCCACTTTGTCCA
ACCACAAGTGAACCCTCTCATAAAGCTCCTTGCAAATGGGATCAATTCCCTGGAAGGAGAA
CAATGGGCACAAAGGAGGAAGATGATCAAACCAGCCTTCCATATTGACAAACTCAAGGAA
ATGGTTCCTACCTTTATAACAAGTTGCAGTGAATTGATTGAGAGGTGGAAGAAGCTACTCAC
TGATGAAACATCTTGTGAATTGGATGTTTGGCCAGAGTTTCAGAATCTAACTATTGATGCGA
TCTCTCGGATCTTGTTTGGGAATAGTTTTGAAGAAGGAAAGCGGATTTTCGAACTCCAAAA
GGAACAAATTGTTCTTGCCATCGATGCCGCCAGCTCTATCTATGTTCCTGGCTTCAGATTCTT
GCCAACTACAAAAAATAAGAGGAGGATGTTCATCGACAATGAGATCAAAAGAATATTGCAA
GATATCATCAACAATAAACTAGAATCAATGAAAATGGGAGGAAATGCGAATAGTGACATACT
CAGCTTGATGTTGCAATACGGTAACATCAACATAGCCGAAAAAGAGAAAAACACAAACAA
CTCCAAGATCACTATCGACAATGTAATCGAGGAGTGCAAAGGCTTCTACTTTGCCGGCCAA
GACACCACCTCGACTCTACTTACTTGGACATTGATTCTCTTATCGATGCACCCTACTTGGCAG
CAAAAAGCAAGGGAAGAAGTTCTTCGAACTTGTGGGAAAAACACACCCAACTTCGAATCC
ATCAACCATCTCAAGATTGTAAACATGATACTACACGAAGTATTAAGATTATATCCACCGGTG
ATCACTCAGATTCGGCACACAGAAAAGAAAACTAAATTAGGAGACATAACATTACCTGCAG
GGGTTCATGTCTTGATACCTACTTTACAAGTTCACCATGATCAAGAATTTTGGGGAGAAGAT
GCAGAGGAGTTTAATCCAGAGAGGTTTTCAGAAGGTGTTTCTAAGGCATCAAAGGGTCAG
AATGCATTCCTTCCATTTGGTTGGGGACCAAGGATGTGTTTAGGGCAAGCATTTGCAATGAT
AGAAACAAAGTTAGGGTTAGCAATGGTGCTTCAACATTTCTCTTTTGAGCTTTCACCTTCTT
ATGCTCATGCTCCTTCAATTGTGGCCACTCTTCAGCCTCAGTTGGGAGCTCATCTCATTCTTC
ATCAACTTTAA

>DzCYP72A17

ATGCTGGAGATGCAGCTGAGAAGACAGGGGTTGCCCGGGAACAAGTATCGGCTGATGATG
GGGGACATGAAGGATGAGAAAAAGTCCTTCAAGGAGGCTTGGTCCAGGCCAATGGAGCTC
ACGCACAGGATAGCGGCTCGTGTGATTCCCTATGATCATCAAATGGCTCAAACACATGGCAA
GATATCATTCAAATGGAATGGAACAACTCCCAGAGTGAACATCTGGAATCCAGAGATGTCG
AGAGAGATCCTGTTGAACAAGTCCGGCCACATTATCAAGCCACAACTTAACCCTCTGATAA
GGCTGCTTACAATGGGAGTATCTACACTTGAAGGAGAAGAATGGGCACAGAGGAGGAAGT
TGATCAATCCTGCCTTTCATATGGAGAAACTCAAGGAGATGGTACCTGTTTTTCGAATCAGT
TGCATTGATTTAGTCAAGAGGTGGGAGAACTTAGTGAGTGCCGAAGGATCCTGTGAACTAG
ATGTCTGGCCTGAATTTCAGAGTCTAACAGGAGATGTGATCTCTCGCACTGCATTTGGTAGT
AGTTTTGAGGAAGGAAAGCAGATTTTTGAACTCCAAAAGGAACAAGCTGTTCTTGTCATTG

AAGCTTCCCGCTCGATCTATCTTCCAGGCTTTAGGTTCCTGCCAACTGCAAAGAATAAGAGG
AGAATGTTCATCGACAGTGAGATCAAAAGAATGCTACGAGATATCATCCACAAGAAGTTAG
ATTCAATGAAAATCGGAGAAAACGCTGATGATGACTTACTCAGCTTGTTGTTGCAATCCGAT
ACGATGAATGTTGTCGCTGAAGATAAAAACAAGAAGAACAATGGGATCACGATTGATGATG
TAATAGAGGAATGCAAGTTGTTCTACTTCGCTGGCCAAGAGGGCACCTCAATTTTGCTTACC
TGGACATTGATTCTCTTATCCATGTACCCTAGTTGGCAGAAGAAAGCAAGGGAGGAAGTTC
TCAATAACTGCGGGAAGAACACACCTGAGTTCGAGAACATCAGCCACCTCAAGATTGTAAA
CATGATATTACATGAAGTATTGAGGTTGTATCCACCTGGGGTCACACTGATTCGTTACATAAA
CAAGAAAGTCAAAGTGGGAAATATAACATTACCTGCTGGAGCTGAAGTCTTGATACCTATTC
TACAAGTACACCATGATCCAGAAATTTGGGGAGAAGATGCTGAAGAGTTCAAACCAGAGA
GATTTTCAGAAGGGGTTTCAAATGCATCAAAGGGTCAGCAAGCATTCTTTCCTTTTGGTTGG
GGCCCCAGAATATGTAGTGGGCAAACTTTCGCTATGATAGAAGCAAAGCTAGCTCTGGCAA
TGGTGCTTCAAAATTTCTCCTTTGAACTCTCACCTTCCTATACTCATGCTCCTTACAATGTGA
TAACCCTTCAGCCACAATATGGAGCTCATCTCATCCTACATCAACTCTGA

>DzCYP72A18

ATGAATGTTGCCACTGAAGATAAAAACAAGAAAAACAATGGGATCACGATTGATGATGTAA
TAGAGGAATGCAAGTTGTTCTACTTCGCTGGCCAAGAGACCACCTCAATTTTGCTTACCTGG
ACATTGATTCTCTTATCCATGTACCCTACTTGGCAGAAGAAAGCTAGGGAGGAAGTTCTCAA
TACCTGTGGGAAGAACACACCTGAGTTCGAGAACATCAGCCACCTCAAGATTGTAAACATG
ATATTACATGAAGTATTGAGGTTGTATCCACCTGTGATCACACTGTTTCGTCACATAAACAAG
AATGTCAAACTGGGAGATATAACATTACCTGCTGGAGCTGAAGTCTTGATACCTATTCTACA
AGTACACCATGATCCAGAAATTTGGGGGGAGGATGCTGAAGAGTTCAAACCAGAGAGATTT
TCAGAAGGGGTTTCAAATGCATCAAAGGGTCAGAATGCATTCTTTCCGTTTGGCTGGGGCC
CAAGAATATTGATAGGTGTTCTAGGGAGAGTGTCATACTTGGTATGGTGGAAGCCAAAAGAT
GCTGGAGATGCAGCTGAGAAGACAGGGGTTGCCAGGAACAAGTATCGGCTGATGATGGGG
GACATGAAGGATGAGAAAAAGTCCTTCAAGGAGGCTTGGTCCAGGCCAATGGAGCTCACG
CACAGGATAGCGGCTCGTGTGATTCCCTATGATCATCAAATGGCTCAAACACATGGCAAGAT
ATCATTCAAATGGAATGGAACAACTCCCAGAGTGAACATATGGAATCCAGAGATGTCGAGA
GAGATCCTGTTAAACAAGTCTGGCCACATTATCAAGCCACAACTTAACCCTCTGATAAGGCT
GCTTACAATGGGAGTATCTACACTTGAAGGAGAAGAATGGGCACAGAGGAGGAAGTTGAT
CAATCCTGCCTTTCATATGGAGAAACTCAAGGAGATGGTACCTGCTTTTCGAATCAGTTGCA
TTGATTTAGTCAAGAGGTGGGAGAACTTAGTGAGTGCCGAAGGATCCTGTGAACTAGATGT
CTGGCCTGAATTTCAGAGTCTAACAGGAGATGTGATCTCTCGCACTGCATTTGGTAGTAGTT
TTGAGGAAGGAAAGCAGATTTTTGAGTTCCAAAAGGAACAAGCTGTTCTTGTCATTGAAGC
TGCCCGCTCGATCTATCTTCCAGGCTTTAGGTTCCTGCCAACTGCAAAGAATAAGAGGAGA
ATGTTCATCGACAGTGAGATCAAGAGAATGCTACGAGATATCATCCACAAGAAGATAGATTC
AATGAAAATCGGAGAAAACACTGATGATGACTTACTCAGCTTGTTTATTTGCAATCCGATAC
TATGA

>DzCYP72A19

ATGTACCCTACTTGGCAGAAGAAAGCTAGGGAGGAAGTTCTCAATACCTGTGGGAAGAAC
ACACCTGAGTTCGAGAACGTCAGCCACCTCAAGATTGTAAACATGATATTACATGAAGTATT
GAGGTTGTATCCACCTGTGATCACACTGTTTCGTCACATAAACAAGAATGTCAAACTGGGA
GATATAACATTACCTGCTGGAGCTGAAGTCGTGATACCTATTCTACAAGTACACCATGATCCA
GAAATTTGGGGAGAAGATGCTGAAGAGTTCAAACCAGAGAGATTTTCAGAAGGGGTTTCA

AATGCATCAAAGGGTCAGAATGCATTCTTTCCTTTTGGTTGGGGCCCAAGAATATGTATTGG
GCAAACTTTCGCTATGATAGAAGCAAAGCTAGCTCTGGCAATGGTGCTTCAACATTTCTCCT
TTGACCTCTCTCCTTCCTATACTCATGCTCCTTACACTGTAATAACCCTTCAGCCACAATATG
GAGCTCATCTCATCCTACATCAACTCTGA

>DzCYP72A20

ATGGGGGTGCTATGGAGAGTGTTATACTTGGTATGGTGGAAGCCAAAGATGCTGGAGATGC
AGCTGAAAAGGCAGGGGTTGCCGGGGAACAAGTATCGGCTGATGATGGGGGACATGAAGG
ATGAGAAAAAGTCCTTCAAGGAGGCTTGGTCCAGGCCAATGGAGCTCACGCACAGGATAG
CGGCTCGTGTGATTCCCTATGATCATCAAATAATGGCTCAAACACATGGCAAGATATCATTCA
AATGGAAAGGGACAACTCCCAGAGTGAACATATGGAATCCAGAGATGTTGAAAGAGATCC
TGTTGAACAAGTCTGGCCACATTATCAAGCCACAAGTGAACCCTCTGATAAAGCTGCTTAC
AATGGGAGTATCTACACTTGAAGGAGAAGAATGGGCACAGAGGAGGAAGTTGATCAATCC
TGCCTTTCATATGGAGAAACTCAAGGAGATGGTACCTGCTTTTCGAATCAGTTGCATTGATT
TAGTCAAGAGGTGGGAGAACTTAGTGAGTGCCGAAGGATCCTGTGAACTAGATGTCTGGCC
TGAATTTCAGAGTCTAACAGGAGATGTGATCTCTCGCACTGCATTTGGTAGTAGTTTTGAGG
AAGGAAAGCAGATTTTTGAACTCCAAAAGGAACAAGCTGTTCTTGTCATTGAAGCTGCCCG
CTCGATCTATCTTCCAGGCTTTAGGTTCCTGCCAACTGCAAAGAATAAGAGGAGAATGTTCA
TCGACAGTGAGATCAAGAGAATGCTACGAGATATCATCCACAAGAAGATAGATTCAATGAA
AATCGGAGAAAATACTGATGATGACTTACTCAGCTTGTTGTTGCAATCCGATACGATGAATG
TTGCCACTGAAGATAAAAACAAGAAAAACAATGGGATCACGATTGATGATGTAATAGAGGA
ATGCAAGTTGTTCTACTTCGCTGGCCAAGAGACCACCTCAATTTTGCTTACCTGGACATTGA
TTCTCTTATCCATGTACCCTACTTGGCAGAAGAAAGCTAGGGAGGAAGTTCTCAATACCTGT
GGGAAAAACACACCTGAGTTCGAGAACATCAGCCACCTCAAGATTGTAAACATGATATTAC
ATGAAGTATTGAGGTTGTATCCACCTGTGATCACACTGTTTCGTCACATAAACAAGAATGTC
AAACTGGGAGATATAACATTACCTGCTGGAGCTGAAGTCTTGATACCTATTCTACAAGTACA
CCATGATCCAGAAATTTGGGGGAGGATGCTGAAGAGTTCAAAACCAGAGAGATTTTCAGA
AGGGGTTTCAAATGCATCAAAGGGTCAGAATGCATTCTTTCCTTTTGGTTGGGGCCCAAGA
ATATGTATTGGGCAAACTTTCGCTATGATAGAAGCAAAGCTAGCTCTGGCAATGGTGCTTCA
ACATTTCTCCTTTGACCTCTCTCCTTCCTATACTCATGCTCCTTACACTGTAATAACCCTTCAG
CCACAATATGGAGCTCATCTCATCCTACATCAACTCTGA

>DzCYP72A21

ATGCTGGAGATGCAGCTGAGAAGACAGGGGTTGCCCGGGAACAAGTATCGGCTGATGATG
GGGGACATGAAGGATGAGAAAAAGTCCTTCAAGGAGGCTTGGTCCAGGCCAATGGAGCTC
ACGCACAGGATAGCGGCTCGTGTGATTCCCTATGATCATCAAATGGCTCAAACACATGGCAA
GATATCATTCAAATGGAATGGAACAACTCCCAGAGTGAACATCTGGAATCCAGAGATGTCG
AGAGAGATCCTGTTGAACAAGTCTGGCCACATTATCAAGCCACAACTTAACCCTCTGATAA
GGCTGCTTACAATGGGAGTATCTACACTTGAAGGAGAAGAATGGGCACAGAGGAGGAAGT
TGATCAATCCTGCCTTTCATATGGAGAAACTCAAGGAGATGGTACCTGTTTTTCGAATCAGT
TGCATTGATTTAGTCAAGAGGTGGGAGAACTTGGTAGTGAGTGCCGAAGGATCCTGTGAAC
TAGATGTCTGGCCTGAATTTCAGAGTCTAACAGGAGATGTGATCTCTCGCACTGCATTTGGT
AGTAGTTTTGAGGAAGGAAAGCAGATTTTTGAACTCCAAAAGGAACAAGCTGTTCTTGTCA
TTGAAGCTGCCCGCTCGATCTATCTTCCAGGCTTTAGGTTCCTGCCAACTGCAAAGAATAAG
AGGAGAATGTTCATCGACAGTGAGATCAAGAGAATGCTACGAGATATCATCCACAAGAAGA
TAGATTCAATGAAAATCGGAGAAAACACTGATGATGACTTACTCAGCTTGTTGTTGCAATCC

GATACTATGAATGTTGCCACTGAAGATAAAAACAAGAAAAACAATGGGATCACGATTGATG
ATGTAATAGAGGAATGCAAGTTGTTCTACTTTGCTGGCCAAGAGACCACCTCAATTTTGCTT
ACCTGGACATTGATTCTCTTATCCATGTACCCTACTTGGCAGAAGAAAGCTAGGGAGGAAGT
TCTCAATACCTTTGGGAAGAACACACCTGAGTTCGAGAACATCAGCCACCTCAAGATTGTA
AACATGATATTACATGAAGTATTGAGGTTGTATCCACCTGTGATCACACTGTTTCGTCACATA
AACAAGAATGTCAAACTGGGAGATATAACATTACCTGCTGGAGCTGAAGTCTTGATACCTAT
TCTACAAGTACACCATGATCCAGAAATTTGGGGGGAGGATGCTGAAGAGTTCAAACCAGAG
AGATTTTCAGAAGGGGTTTCAAATGCATCAAAGGGTCAGAATGCATTCTTTCCTTTTGGTTG
GGGCCCAAGAATATGTATTGGGCAAACTTTCGCTATGATAGAAGCAAAGCTAGCTCTGGCA
ATGGTGCTTCAACATTTCTCCTTTGACCTCTCTCCTTCCTATACTCATGCTCCTTACACTGTAA
TAACCCTTCAGCCACAATATGGAGCTCATCTCATCCTACATCAACTCTGA

>DzCYP72A22

ATGCTGGAGATGCAGCTGAGAAGACAGGGGTTGCCGGGGAACAAGTATCGGCTGATGATG
GGGGACATGAAGGATGAGAAAAAGTCCTTCAAGGAGGCTTGGTCCAGGCCAATGGAGCTC
ACGCACAGGATAGCGGCTCGTGTGATTCCCTATGATCATCAAATGGCTCAAACACATGGCAA
GATATCATTCAAATGGAATGGAACAACTCCCAGAGTGAACATCTGGAATCCAGAGATGTCG
AGAGAGATCCTGTTGAACAAGTCTGGCCACATTATCAAGCCACAACTTAACCCTCTGATAA
GGCTGCTTACAATGGGAGTATCTACACTTGAAGGAGAAGAATGGGCACAGAGGAGGAAGT
TGATCAATCCTGCCTTTCATATGGAGAAACTCAAGGAGATGGTACCTGTTTTTCGAATCAGT
TGCATTGATTTAGTCAAGAGGTGGGAGAACTTAGTGAGTGCCGAAGGATCCTGTGAACTAG
ATGTCTGGCCTGAATTTCAGAGTCTAACAGGAGATGTGATCTCTCGCACTGCATTTGGTAGT
AGTTTTGAGGAAGGAAAGCAGATTTTTGAACTCCAAAAGGAACAAGCTGTTCTTGTCATTG
AAGCTTCCCGCTCGATCTATCTTCCAGGCTTTAGATTCCTGCCAACTGCAAAGAATAAGAGG
AGAATGTTCATCGACAGTGAGATCAAAAGAATGCTACGAGATATCATCCACAAGAAGTTAG
ATTCAATGAAAATCGGAGAAAACGCTGATGATGACTTACTCAGCTTGTTGTTGCAATCCAAT
ACGATGAATGTTGCCGCTGAAGATAAAAACAAGAAGAACAATGGGATCACGATTGATGATG
TAATAGAGGAATGCAAGTTGTTCTACTTCGCTGGCCAAGAGGGCACCTCAATTTTGCTTACC
TGGACATTGATTCTCTTATCCATGTACCCTAGTTGGCAGAAGAAAGCAAGGGAGGAAGTTC
TCAATAACTGCAGGAAGAACACACCTGAGTTCGAGAACATCAGCCACCTCAAAATTGTAAA
CATGATATTACATGAAGTATTGAGGTTGTATCCACCTGGGGTCACACTGATTCGTTACATAAA
CAAGAAAGTCAAAGTGGGAAATATAACATTACCTGCTGGAGCTGAAGTCTTGATACCTATTC
TACAAGTACACCATGATCCAGAAATTTGGGGAGAAGATGCTGAAGAGTTCAAACCAGAGA
GATTTTCTGAAGGGGTTTCAAATGCATCAAAGGGTCAGCAAGCATTCTTTCCTTTTGGTTGG
GGCCCCAGAATATGTAGTGGGCAAACTTTCGCTATGATAGAAGCAAAGCTAGCTCTGGCAA
TGGTGCTTCAAAATTTCTCCTTTGAACTCTCACCTTCCTATACTCATGCTCCTTACAATGTGA
TGACCCTTCAGCCACAATATGGAGCTCATCTAATCCTACATCAACTCTGA

>DzCYP72A23

ATGGAGTCAGTGATGGGAGTGATATGGGCGGTGGCGGCGGTGGTGGTAGTGGCGGCGGCG
TGGAGGACATTGGATTGGATTTGGTGGACACCGAGGAGGCTGGACCGGGAGCTCCGGCGG
CAGGGCCTGCGCGGCAACCAGTACCGAGTCTTGCACGGCGATCTCAAGGAAAACGTCCGG
CTCTCGAAGGAGGCTAAATCCCGGCCTCTGCCTCTTCACTGCCATGACATCGCCCCCCGCGT
TCTCCCTCTCTTCCACAACGCCATCAAAGATCACGGTAAAATCTCAATAACTTGGCTTGGCC
CTTATCCAAGAGTGACCTTAACAGAGCCAGAGCTAGTAAAAGAAGTGCTGTCAAACAAGTT
TGGACATTTTGTTAAACCAACTACAAATCCCTTAGCCAAATTTTTGATCCAAGGGCTTGCGT

CTTATGAAGGTGAAAAGTGGGCCAAACACAGAAGGATCATCAACCCTGCATTCCATCTTGA
GAAACTAAAGCTAATGCTGCCAGCATTCTCTACATCTTGTGGTGAACTGATTAGAAGATGGG
AGAAGATGATTCCTAATGAAGGCTCCCAAGAACTAAATGTCTTTCCAGAGCTCCAAGACCT
CACAAAAGATGTCATCTCCAGGACTGCATTCGGTAGCAGTTATGAAGACGGAAGAAGAATA
TTTGAACTCCTAACAGAGCAAATTCAGCTTCTTATTCCAGCTTTCCAGACTGTATACATTCCT
GGTTATCGATTTCTGCCCACACCAATGAACAAAAGAAGCAGCCAAGTGTACAATGAGATGA
AAAGAATTCTTATAGGCATGATTGAGAAGAGAGAAAAGGCCATAAGAATGGGGGAAAGTA
GCAAGAATGACCTTCTGGGTTTGTTACTAGACTCCAATATCAAAGAGAGTGAAGTGCATGG
GAAGTCCCAAAACAAAGGGATGAGCACTGAGGATGTGGTTGAAGAGTGCAAGCTGTTCTA
CTTTGCAGGGCAAGAGACTACATCACTTCTACTCACATGGACAATGATTTTATTGAGCATGT
ATCCAAATTGGCAGGCTAAGGCAAGAGAAGAGGTTCTTCAAGTCTTTGGAAAGAGCGCAC
CAGATATGGAGGGATTGAGCCACTTGAAGATTGTGACCATGATTCTATATGAAGTTCTAAGG
TTATATCCACCAGCGGTTTTCCTAACAAGAAAAACCTACAAAGCAATGGAACTCGGTGGAA
TTACTTACCCTCCAGGAGTGATACTCTCACTGCCTCTACTCTTCATTCACCATGACCCTGTTT
TCTGGGGAGAAGACGCCAAAGAGTTTAATCCAGAGAGGTTTTCCGAAGGGATATCGAAAG
CATCCAAAGTTCCGGGTGCCTTCTTTCCTTTCGGTGGAGGTCCGCGCATTTGCATTGGCCAA
AACTTTGCTCTGATTGAAGCTAAGATAGGAATTTGCATGATTCTTCAGCACTTCTCCTTTGTG
CTTTCGCCTTCCTATATCCATGCACCGCACAATGTTATTACTCTTCAACCACAGCATGGAGCT
CAACTCATGCTGCAAAAGGTGTGA

>DzCYP72A24

ATGGAGTTAGTGATGGGAGTGATATGGACGGTGACGGCGGCGGTGGTGGTGGTGTGGGCGT
GGAGGACGTTGGATTGGGTTTGGTGGACGCCGAGGAGGCTGGACCGGGATCTCCGGCGCC
AGGGCCTGCGCGGCAACCAGTACCGATTATTGCACGGCGATCTCAAGGAAAACGCCCGGCT
CTCCAAGGAGGCTACATCCCGGCCTTTGCCTCTTCACTGCCATGAAATCGCTCCCAGGGTTG
CCCCTCTCATCCACAACGCCATCAAAGATCACGGTAAAATATCAATAACTTGGCTTGGACCT
TATCCAAGAGTGTCCTTGATGGACCCAGATTTAGTGAAAGAAGTACTATCAAACAAGTTTG
GACATTTTGTTAAACCAAGAATAACTCCTATTGCCAAATTTTTGGTCCAAGGACTTGTAGCT
TATGAAGGTGAAAAGTGGGCCAAACACAGAAGGATAATTAACCCCGCATTCCATCTTGAGA
AACTAAAGCTAATGCTGCCAGCATTCTCTACATCTTCTGGTGAACTGATTAGAAGATGGGAG
AAGATGATCCCTGATGAAGGCTCCCAAGAACTAAATGTCTCGCCAGAGATCCAAAACCTCA
CAGGAGATGTCATCTCCAGGACTGCATTCGGTAGCAGCTATGAAGAAGGGAGAAGAATATT
TGAACTCCTAACAGAGCAAATTCATCTTACTATTCCAGCTTTGCAGACTGTATACATCCCTGG
TTATCGGTTTCTGCCCACACCAATGAACAACAGAAGAAGCCAAGTTTACAATGAGATGAAA
AGAATTCTTAAAGGCATGATTGAGAAGAGAGAGAAGGCCATAAGAATGGGGGAAAGTAGC
AAGAATGACCTTCTGGGTTTGTTACTAGACTCCAATATGAAGGAGGGTGAAGAGCAGGAGA
AGTCCCAAAACAAAGTGATGAGCACTGAGGATGTGATTGAAGAGTGCAAGCTGTTCTACTT
TGCAGGGCAAGAGACTACATCAGTTCTACTCACATGGACAATGATTTTATTGAGCATGTATC
CTAACTGGCAGGCCAAGGCAAGAGAAGAGGTTCTTCAAGTCTTTGGAAAGAGCACACCAG
ACATGGATGGATTGAGCCGCTTGAAGATTGTGACCATGATTCTGTATGAAGTTCTGAGGTTA
TATCCACCGGTGACTTTCCTAACAAGAAAAACATACAAAGCAATGGAACTTGGTGGGATCA
ATTACCCTCCAGGAGTGTTACTCCAACTGCCTCTACTCTTCATTCACCATGACCCTGAATTCT
GGGGAGAAGACGCCAAAGAGTTCAATCCCGAGAGGTTTTCTGAAGGGATATTGAAAGCAT
CCAAAGTTCCGGGTGCCTTCTTTCCTTTCGGTGGAGGTCCGCGCATTTGCATTGGCCAAAG
CTTTGCACTGATTGAAGCTAAGATTGGAATTAGCATGATTCTTCAGCACTTCTCCTTCGAGC

TCTCGCCTTCGTATATCCATGCACCGCACACTCCTCTTACTCTTCAACCACAGCATGGAGCT
CAACTCATGCTTCAAAAACTCTGA

>DzCYP72A25

ATGGAGTACTCAGTGGCGGCAGGGGTGAAGGAGATGATATGGGGTGTGGCAACGGCGCTG
TTGGTGGTGTGGGTGTGGAGGACGTTGGAGTGGTTGTGGTGGAAGCCGAGGAGGCTGGAG
CGCGAGCTGAAGAGGCAGGGATTGCGCGGAGGCAAGTACCGTCTCTTCCACGGCGATCTC
AAGGACAACGCGCGACTCATGAAGGATGCTCTTTCTCGCCCCTTGCCCCCTTACTCTCACG
ACGTCGCCCAACGAGTCATCCCTCTCGTCCATCAAGCTATCAAGATTCATGGTAAAATGTCT
ATAACATGGCTTGGGCCTTATCCTAGAGTGAGCTTGATGGACCCAGAGTTAATAAGAGAAGT
TCTATCAAACAAGTTTGGCCACTTTGTGAAGCCAAACTTCAGTCCCCTTGTGAAACTGTTA
GCCCAAGGGCTTGCAAGTCATGAAGGTGAGAAGTGGGCTAAACATAGGAGGATCATCAAC
CCTGCTTTCCATTTTGAAAAACTAAAGTGCATGTTGCCGGCTTTTTCGACATGTTGTGATGA
ACTGGTTAAGAGATGGCAGAACAAGATGAATGTAGAGGGTTCCATTGAGCTGAATATGTGG
CCAGAGCTCCAAAATCTTACTGGAGATGTCATCTCCAGGACTGCATTTGGTAGTTGCTACGA
AGAAGGAAGAAGAATTTTTCAACTCCAAACAGAGCAAGCTGAACTTATTATTCCAACCGCT
CAGACTGTATATGTTCCTGGTTTCAGGTTTTTACCAACACCAAAGAACAACAGAAGAAAAG
CAATTGATAGAGAGATTAAAACAATTCTGAGAAGCATGATTGAAAAAAGAGAGAAGGCCAT
AAGAATGGGAGGAGAAACCAGCTGCAAGGATGACCTTCTGGGTTTGCTATTGGAGTCCAA
CATGAAGGAGACTGAACAACAAGGCAGGTCCAAAAACAAAGGGCTGACTACTGAGGACG
TGATTGAAGAATGCAAGCTCTTCTACTTAGCAGGGCAAGAGACCACATCATCTCTTCTCACA
TGGACAATGGTTTTGTTGAGCATGTATCCAAACTGGCAGGCTAAAGCCAGAGAAGAAGTTC
TCCAAGTCTTTGGGAAAAACAAACCCGACATGGACGGCGTCGGCCGCCTGAAGATCGTGA
CGATGATACTGTATGAGGTTCTCAGATTATATCCACCAGCCATTTTCCTGACAAGAAGAACAT
ACAAAACAATGGAACTGGGAGGGATAACTTATCCTCCAGGGGTGCTGCTCTTGTTACCCAT
AATCTTGGTTCACCATGACTTGGATTTCTGGGGAGAAGATGCCAAAGACTTTAAGCCGGAT
AGGTTTGCAGAAGGGATATCAAAGGCATCCAAAGTTCCAGGAGCTTTCTTTCCATTTGGTG
GAGGTCCACGTATATGCATTGGCCAAAGCTTTGCACTCATTGAAGCTAAGCTGGGACTTAGC
ATGATTCTTCAGAACTTCTCCTTTGAGCTCTCACCTTCTTATATCCATGCTCCTTACACTCTTG
TTACTCTTCAGCCTCAGCATGGAGCTCCAATCAAGCTGCACAAACTCTGA
