# Supplementary material for: Genome-Wide Identification of CYP72A Gene Family and Expression Patterns Related to Jasmonic Acid Treatment and Steroidal Saponin Accumulation in Dioscorea zingiberensis
Source: Int J Mol Sci. 2021 Oct 11;22(20):10953. doi: 10.3390/ijms222010953 (PMC8536171; doi:10.3390/ijms222010953)
Supplement: Supplementary file 1 [file ijms-22-10953-s001.zip › Table S4 Table S4 Primer sequences used for quantitative real-time PCR.pdf]

Table S4 Primer sequences used for quantitative real-time PCR

Name	**Forward primer Sequence (5' →3')**	**Reverse primer Sequence (5' →3')**
DzCYP72A1	GTTTGGTGGAAGCCGAGGAG	ATCTTTGATGGCGTTGTGGA
DzCYP72A2	AACCTACAAAGCAATGGAACTCG	AAAAGCAGGGTCATGGTGAAT
DzCYP72A3	CCCAAGACAAAGGGATGAGC	GCCTGCCAGTTCGGATACA
DzCYP72A4	ATGTATCCGAACTGGCAGACTAA	GGTCACAATCTTCAAGTGGCTC
DzCYP72A5	AGCAATGGAACTCGGTGGG	AAAAGCAGGGTCATGGTGAAT
DzCYP72A6	CCCAAGACAAAGGGATGAGC	GCCTGCCAGTTCGGATACA
DzCYP72A7	ATGTATCCGAACTGGCAGACTAA	GGTCACAATCTTCAAGTGGCTC
DzCYP72A8	AAGCATCCAAAGTTCCAGGTG	GCTTTTGCAGCATGAGTTGAG
DzCYP72A9	GGGATTGAACCACTTGAAGATTG	CACCGAGTTCCATTGCTTTGTA
DzCYP72A10	CCGAACTGGCAGGCTAATG	TCAAGTGGCTCAATCCCTCTAT
DzCYP72A11	CCCAAAACAAAGGGATGAGC	GCCTGCCAGTTCGGATACA
DzCYP72A12	CGGTAGCAGTTACGAAGACGG	GCAGAAATCGAAAACCAGGAA
DzCYP72A13	ACCCCGCATTCCATCTTG	AGACATTTAGTTCTTGGGAGCCT
DzCYP72A14	TGGCAGAACAAGATGAATGTAGAG	ATACAGTCTGAGCGGTTGGAATA
DzCYP72A15	GCTGACCTGGACACTGATACTTTT	TGGCATGTTCTTCCCACAAAT
DzCYP72A16	AGGCATCAAAGGGTCAGAATG	CCCAACTGAGGCTGAAGAGTG
DzCYP72A17	TCTATCTTCCAGGCTTTAGGTTCC	TCAGCGTTTTCTCCGATTTTC
DzCYP72A18	GAGGATGCTGAAGAGTTCAAACC	CCCAGCCAAACGGAAAGA
DzCYP72A19	TACCTGCTGGAGCTGAAGTCG	GCCCCAACCAAAAGGAAAG
DzCYP72A20	ACGATGAATGTTGCCACTGAA	TGCCAAGTAGGGTACATGGATAA
DzCYP72A21	TTGGGCAAACTTTCGCTATG	AAGGGTTATTACAGTGTAAGGAGCA
DzCYP72A22	TGGCAGAAGAAAGCAAGGG	TCAGCTCCAGCAGGTAATGTTATA
DzCYP72A23	GGTGCCTTCTTTCCTTTCGG	AGAGTAATAACATTGTGCGGTGC
DzCYP72A24	ACCCCGCATTCCATCTTG	AGACATTTAGTTCTTGGGAGCCT
DzCYP72A25	CACGGCGATCTCAAGGACA	GCTAACAGTTTCACAAGGGGAC
DzActin	ATGCCATTCTTCGTTTGGAC	CTACTCTTGGCGGTTTCCAG
DzGAPDH	TTTGGTGAAAAGTCAGTCGCA	TCGGAGCAGAAATGACAACCT
