# Supplementary material for: Genome-Wide Identification of CYP72A Gene Family and Expression Patterns Related to Jasmonic Acid Treatment and Steroidal Saponin Accumulation in Dioscorea zingiberensis
Source: Int J Mol Sci. 2021 Oct 11;22(20):10953. doi: 10.3390/ijms222010953 (PMC8536171; doi:10.3390/ijms222010953)
Supplement: Supplementary file 1 [file ijms-22-10953-s001.zip › Table S5 DCA analysis of specialized metabolites in the biosynthesis of steroidal saponins.pdf]

Table S5 DCA analysis of specialized metabolites in the biosynthesis of steroidal saponins

Statiscs	DCA1	DCA2
Eigenvalues	0.0256	0.0076
Decorana values	0.0267	0.0004
Axis lengths	0.3588	0.1952
