# Supplementary material for: Genome-Wide Identification of CYP72A Gene Family and Expression Patterns Related to Jasmonic Acid Treatment and Steroidal Saponin Accumulation in Dioscorea zingiberensis
Source: Int J Mol Sci. 2021 Oct 11;22(20):10953. doi: 10.3390/ijms222010953 (PMC8536171; doi:10.3390/ijms222010953)
Supplement: Supplementary file 1 [file ijms-22-10953-s001.zip › Table S6 The constrained result of RDA analysis.pdf]

Table S6 The constrained result of RDA analysis

Axis	Eigenvalue	Proportion Explained	Cumulative Proportion
RDA1	10157.5746	0.7976	0.7976
RDA2	1490.9177	0.1171	0.9146
RDA3	873.4672	0.0686	0.9832
RDA4	168.9920	0.0133	0.9965
RDA5	31.5857	0.0025	0.9990
RDA6	9.5267	0.0007	0.9997
RDA7	2.9864	0.0002	1.0000
RDA8	0.4818	0.0000	1.0000
