# Supplementary material for: Genome-Wide Identification of CYP72A Gene Family and Expression Patterns Related to Jasmonic Acid Treatment and Steroidal Saponin Accumulation in Dioscorea zingiberensis
Source: Int J Mol Sci. 2021 Oct 11;22(20):10953. doi: 10.3390/ijms222010953 (PMC8536171; doi:10.3390/ijms222010953)
Supplement: Supplementary file 1 [file ijms-22-10953-s001.zip › Table S7 Quantitative determinations of each specialized metabolites.pdf]

Table S7 Quantitative determinations of each specialized metabolites

Compound name	Regression equation	r^2
Diosgenin	$y=5*10^{-7}x+1.6219$	0.9937
Parvifloside	$y=8*10^{-8}x-47.3690$	0.9991
Protodeltonin	$y=8*10^{-8}x-61.2890$	0.9972
Dioscin	$y=2*10^{-7}x-0.0007$	0.9755
Cholesterol	$y=8*10^{-8}x+0.6829$	0.9960
Campesterol	$y=6*10^{-8}x+2.0574$	0.9965
Stigmasterol	$y=3*10^{-8}x+0.9850$	0.9977
β-sitosterol	$y=4*10^{-8}x+1.7157$	0.9987

Note: y, the content of specialized metabolites; x, peak area; r^2, regression coefficient
